# Supplementary material for: Reversible gene silencing through frameshift indels and frameshift scars provide adaptive plasticity for Mycobacterium tuberculosis
Source: Nat Commun. 2021 Aug 4;12:4702. doi: 10.1038/s41467-021-25055-y (PMC8339072; doi:10.1038/s41467-021-25055-y)
Supplement: Supplementary file 1 — Supplementary Information [file 41467_2021_25055_MOESM1_ESM.pdf]

Reversible gene silencing through frame-shift indels and frame-shift scars provide adaptive plasticity for *Mycobacterium tuberculosis*

Supplementary Information

Aditi Gupta and David Alland

Supplementary Figures

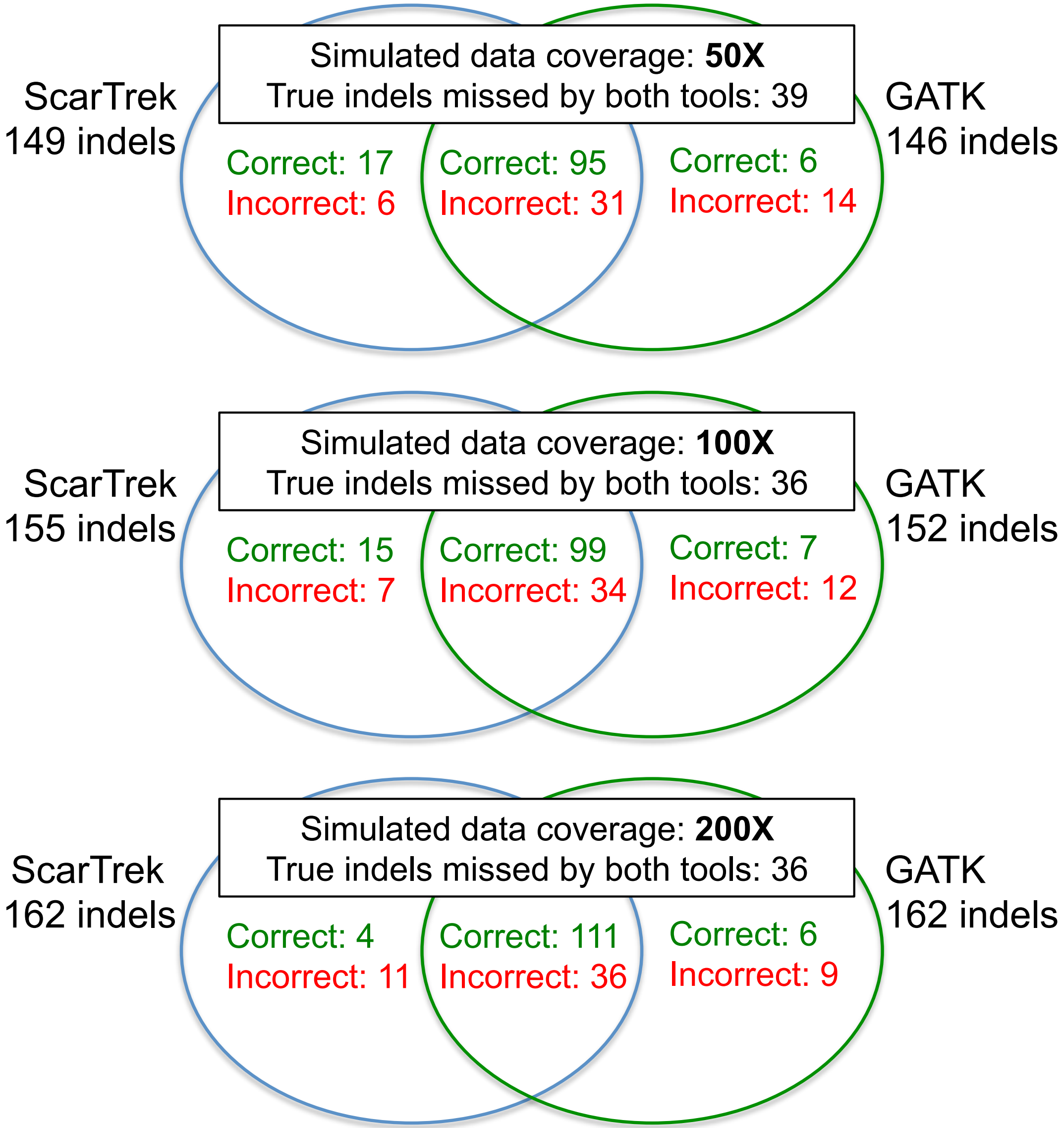

Supplementary figure S1. Indels called using ScarTrek and/or GATK HaplotypeCaller from the simulated WGS of 10 *M. tuberculosis* in which 157 indels were artificially introduced. The Venn diagrams for indel calling from ScarTrek and GATK HaplotypeCaller show that with increasing coverage, the indels correctly detected by both the tools increase from 95 at 50X to 111 at 200X. At low coverages, ScarTrek detects more indels correctly, while GATK HaplotypeCaller minimizes incorrect calls with increasing coverage.

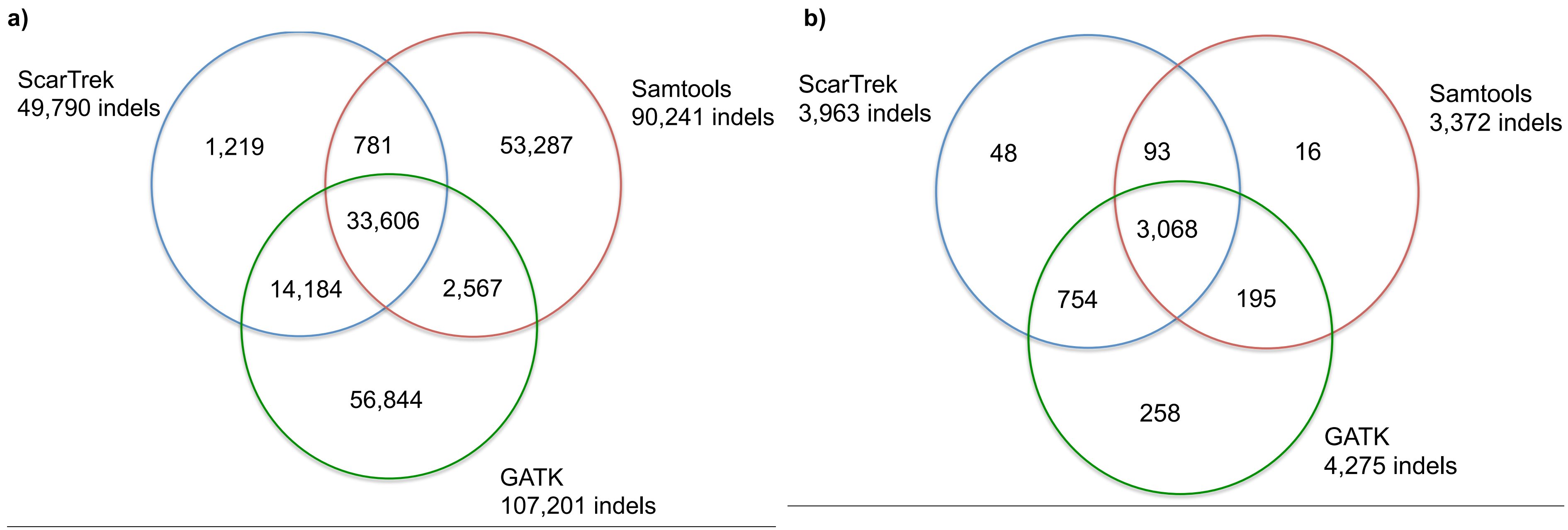

Supplementary figure S2. Indels called using ScarTrek, SAMtools, and GATK HaplotypeCaller for the 402 clinical isolates of *M. tuberculosis*. Venn diagram for indels calls from three methods (ScarTrek, GATK HaplotypeCaller and SAMtools) are shown for (a) all indels present in the clinical isolates. 67% of the indels called by ScarTrek were supported by both SAMtools and GATK HaplotypeCaller. In contrast, only 37% of SAMtools indels and 31% of GATK HaplotypeCaller indels were supported by the other two methods. Only 2.4% (1219 out of 49790) of ScarTrek indels were not supported by any of the other two methods, in contrast to 59% of SAMtools indels and 53% of GATK HaplotypeCaller indels. Panel (b) shows prediction performance for 155 scar indels present in the scar isolates (each occurrence of the 155 scar indels is counted). SAMtools detected the fewest of these indels (3372), followed by ScarTrek (3963) and GATK HaplotypeCaller (4275). The majority of indels were detected by all three tools.

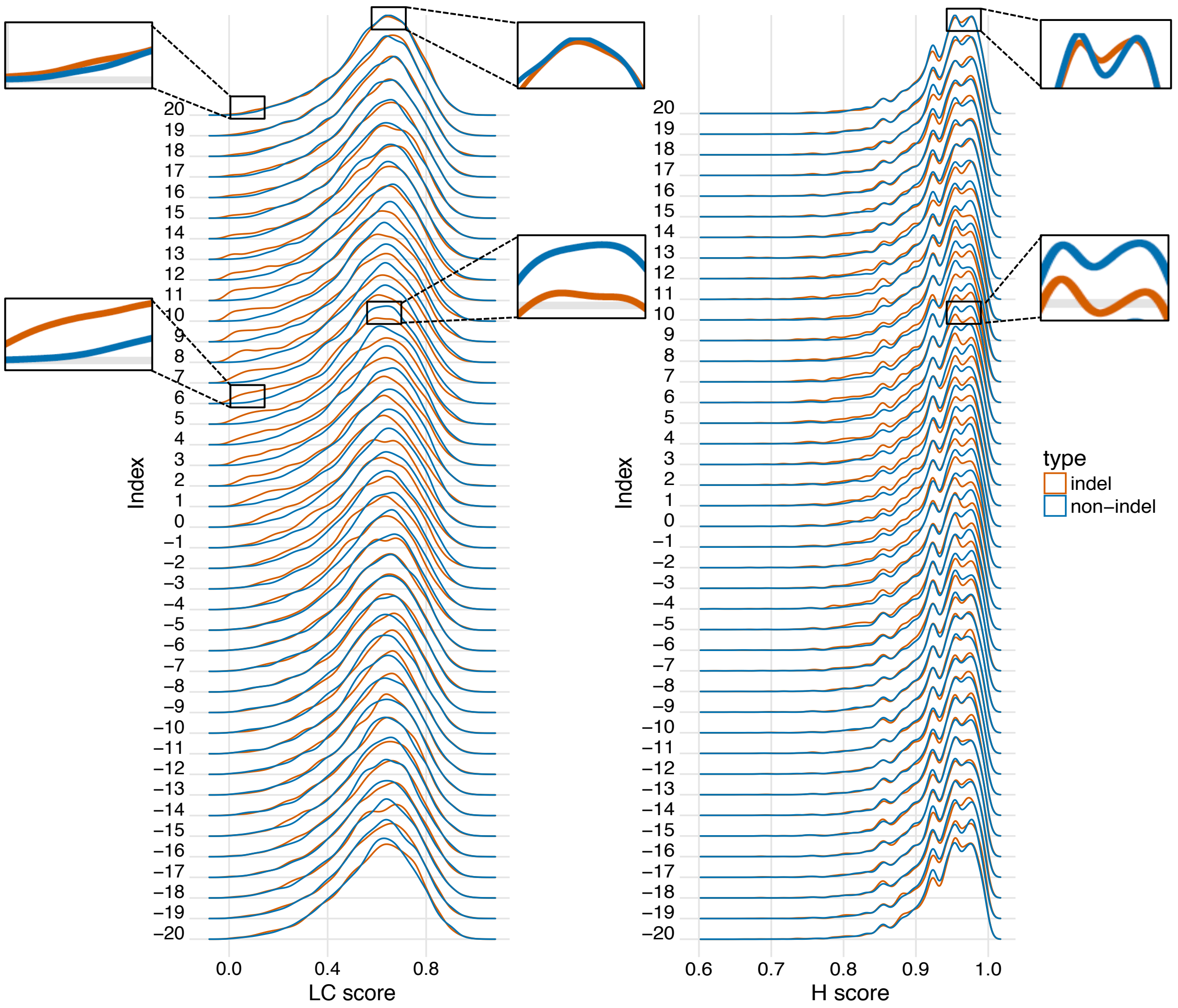

Supplementary figure S3. Sequence complexity around an orphan indel. The histograms of linguistic complexity (*LC* score, panel a) and Shannon's entropy (*H* score, panel b) are shown for 20 positions upstream (y-axis: index -1 to -20) and 20 positions downstream (index 1 to 20) of 5172 orphan indel positions and 5775 non-indel positions (index=0 in y-axis). Orphan indel positions are defined as those that do not have another indel within the 100 bases on either side but have an indel at index=0. The non-indel positions are defined as those that do not have an indel at index=0 and do not have any indels in the 100 bases on either side of index=0. Distributions of *LC* and H scores for indel positions and their neighboring sites are shown in orange, and those for non-indel positions are shown in blue. Parts of the distributions have been enhanced to show the existence of lower complexity scores in the vicinity of indel positions compared to non-indel positions. See Supplementary Datasets 1 and 2 for *LC* scores at 20 sites upstream and downstream of indel and non-indel positions respectively, and Supplementary Datasets 3 and 4 for H scores at 20 sites upstream and downstream of indel and non-indel positions respectively.

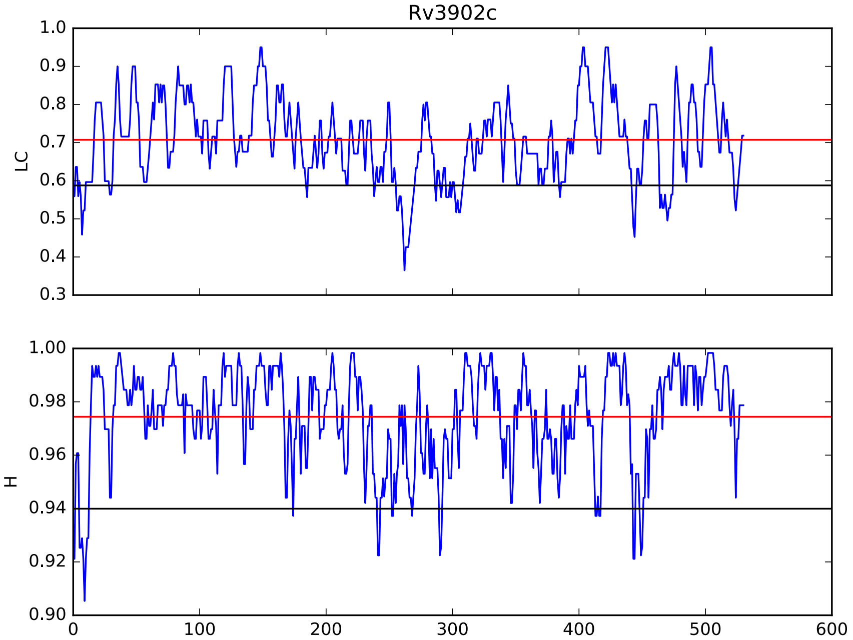

Supplementary figure S4. Sequence complexity profiles for the essential gene *Rv3902c*. Rv3902c has the highest mean complexity scores of all *M. tuberculosis* genes in our analysis. Sequence complexity (*LC:* linguistic complexity, *H*: Shannon's entropy) is shown along the gene length (x-axis). Black lines denote the genomic average of the complexity scores and red lines indicate the genic average of these scores for the entire gene. Note that regions with sequence complexity well below the genome average were present even within this gene.

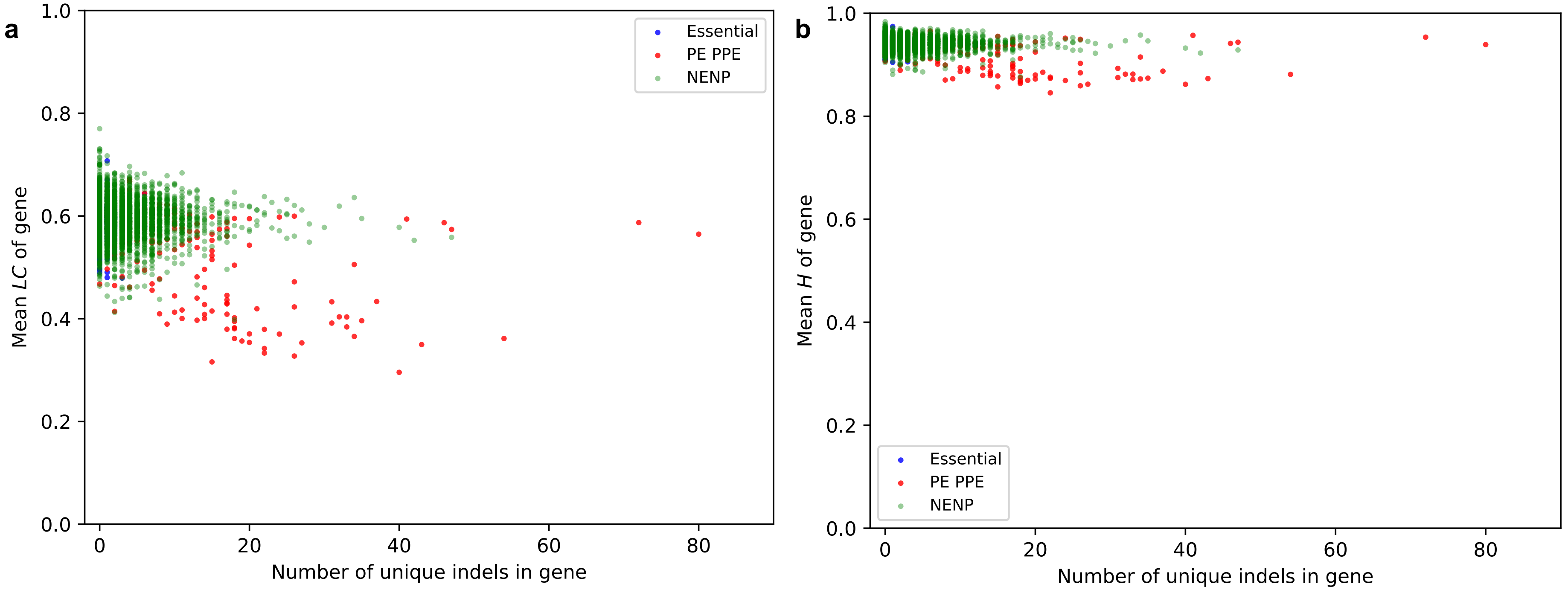

Supplementary figure S5. The mean complexity scores of genes as a function of unique indels. Mean *LC* complexity scores (panel a); and mean *H* complexity scores (panel b) are shown for essential (blue), PE-PPE (red), and remaining non-essential and non-PE-PPE genes (NENP, faded green).

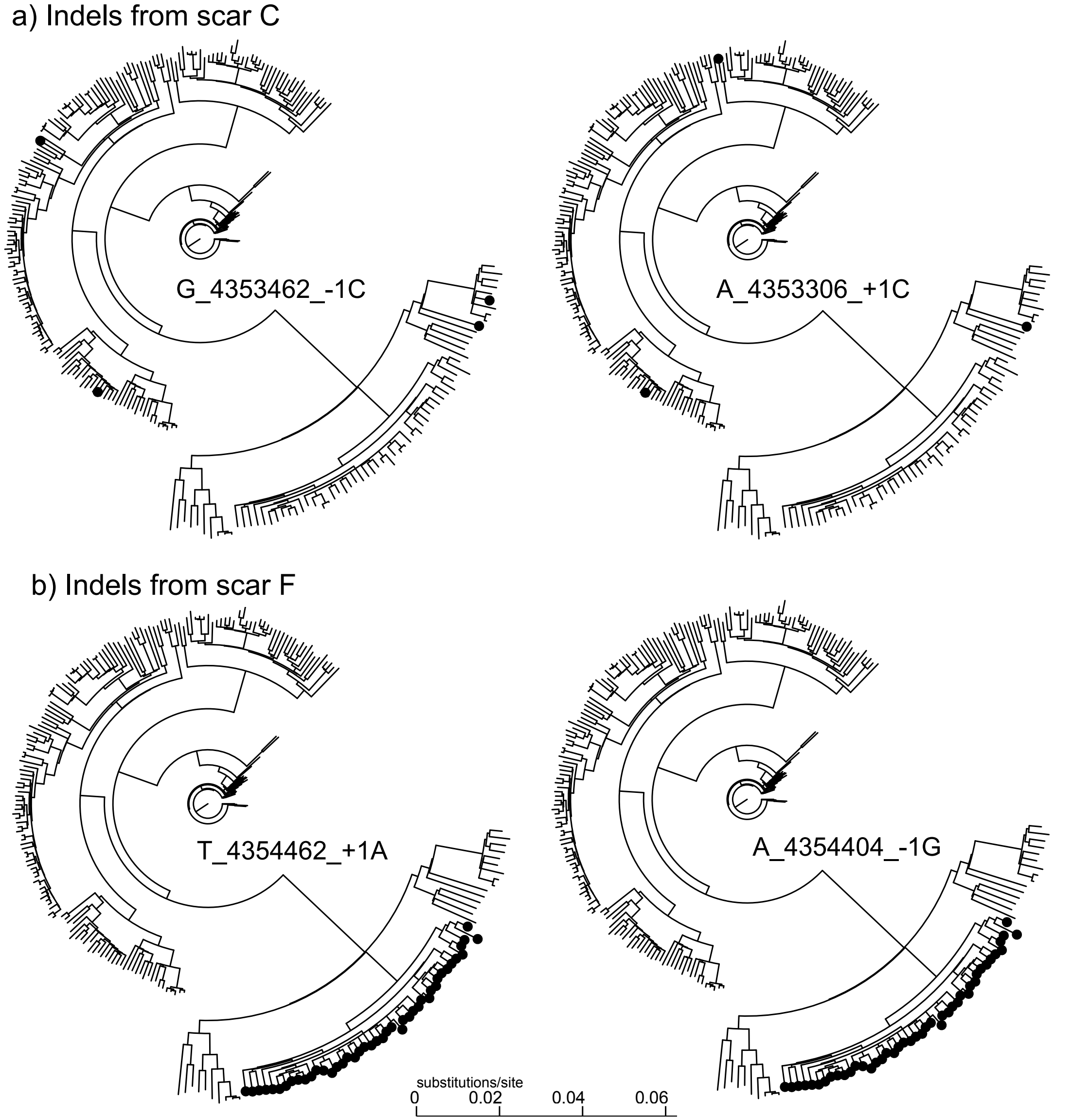

Supplementary figure S6. Phylogenetic relationship of scar indels in gene *espI.* (a) Phylogenetic trees showing isolates that have the indels belonging to scar C (green) in Figure 6 in manuscript. The two indels are A_4353306_+1C and G_4353462_-1C and appear in phylogenetically distant isolates in the tree, suggesting evidence of convergent evolution (isolates containing scar indels are denoted by black circles). (b) Phylogenetic trees showing isolates that have the indels belonging to scar F (gray) in Figure 6 in manuscript. The two indels are A_4354404_-1G and T_4354462_+1A and are present in evolutionarily close isolates, suggesting that these indels were derived from a common ancestor. Although unrooted, the Newick tree visualizations are implicitly rooted at the top node.

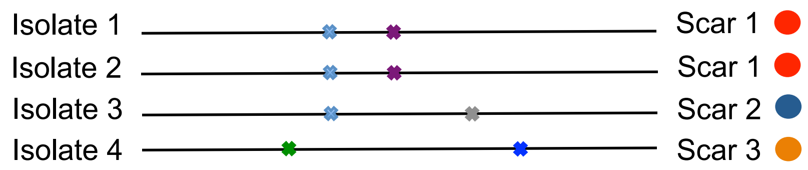

Supplementary figure S7a. Phylogenetic relationship between scars and scar-indels: evidence of convergent evolution or derivation from common ancestor. Schematic of frame-shift scars with two common indels (scar 1, red), with one common indel (scars 1 and 2, red and blue), or with no common indels (scar 3). Genes containing scars are represented by a black line. Each indel is shown as a cross, and different indels are indicated by different colors. This color scheme is followed in panel b-d: identical scars are shown in same color, but scars where one or both indels differ are shown in different colors.

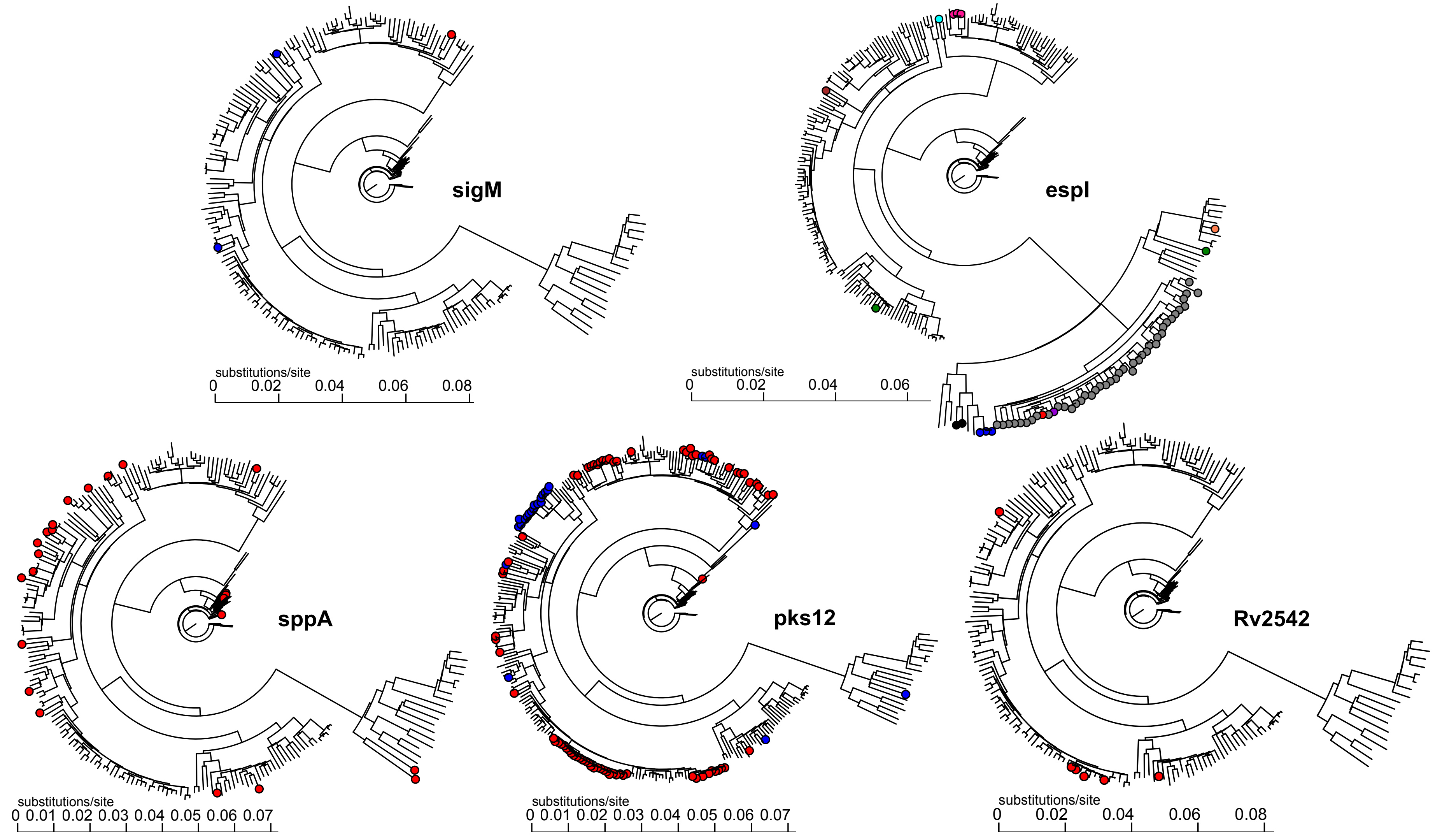

Supplementary figure S7b Convergent evolution of frame-shift scars ("scar 1" in S7a). Each phylogenetic tree depicts the evolutionary relationships between isolates that had a scar in a given gene against a background of 200 non-scarred phylogenetically distant isolates. Scars in six genes (circles with identical colors denote identical scars) restored gene function using the same set of indels in phylogenetically distant isolates (scar 1 scenario in Fig. S7a). Evolutionary unrelatedness of isolates that contain the same scar suggests that these scars evolved independently. Thus, 6 scars likely appeared by convergent evolution by both Bayesian and Neighbor Joining (NJ) methods: one scar each in *Rv2542* (red circles), *sppA* (red circles), *sigM* (blue circles), *espI* (scar denoted by green circles only), and two scars in *pks12* (red and blue circles). NJ trees further identified two scars in *eccE1* (not shown) that were not supported by Bayesian analysis. Although unrooted, the Newick tree visualizations are implicitly rooted at the top node.

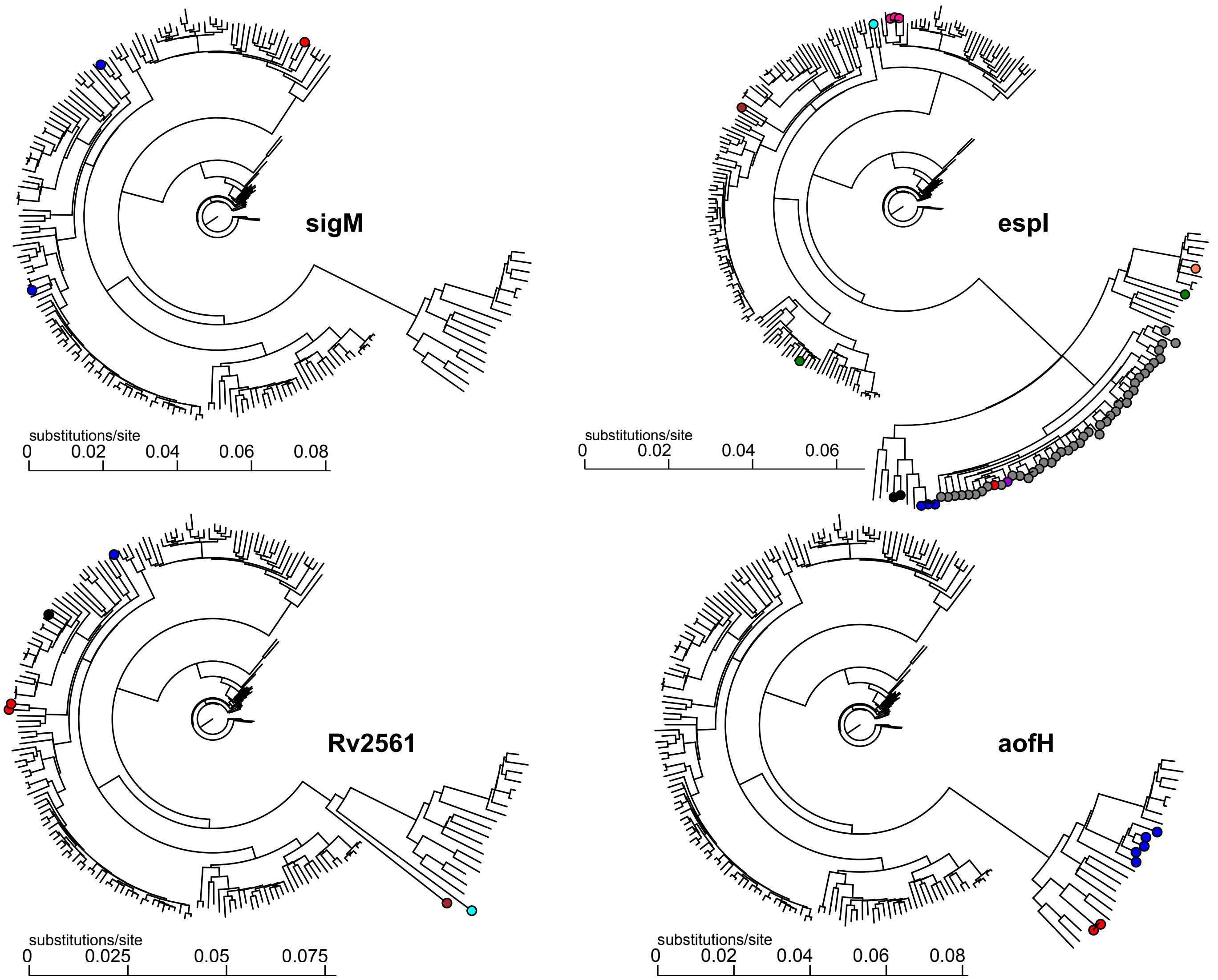

Supplementary figure S7c Convergent evolution of scar indels (scenario depicted by scars 1 AND 2 in S7a). Phylogenetic trees depicting the evolutionary relationships between isolates that had a scar in a given gene against a background of 200 non-scarred phylogenetically distant isolates. Each colored circle denotes a unique scar in a tree. Genes *sigM,* and *aofH* have two scars each (red and blue circles) that share an indel. *Rv2561* has five distinct scars that all share one common indel (shown in colors red, black, maroon, cyan, and blue). In gene *espI,* three scars (coral, brown, and green circles) share an indel. These scar indels likely appeared by convergent evolution (scenario of scar 1 and 2 in Fig. 7a). Convergent evolution of these scar indels was supported by both Neighbor Joining and Bayesian methods. Although unrooted, the Newick tree visualizations are implicitly rooted at the top node.

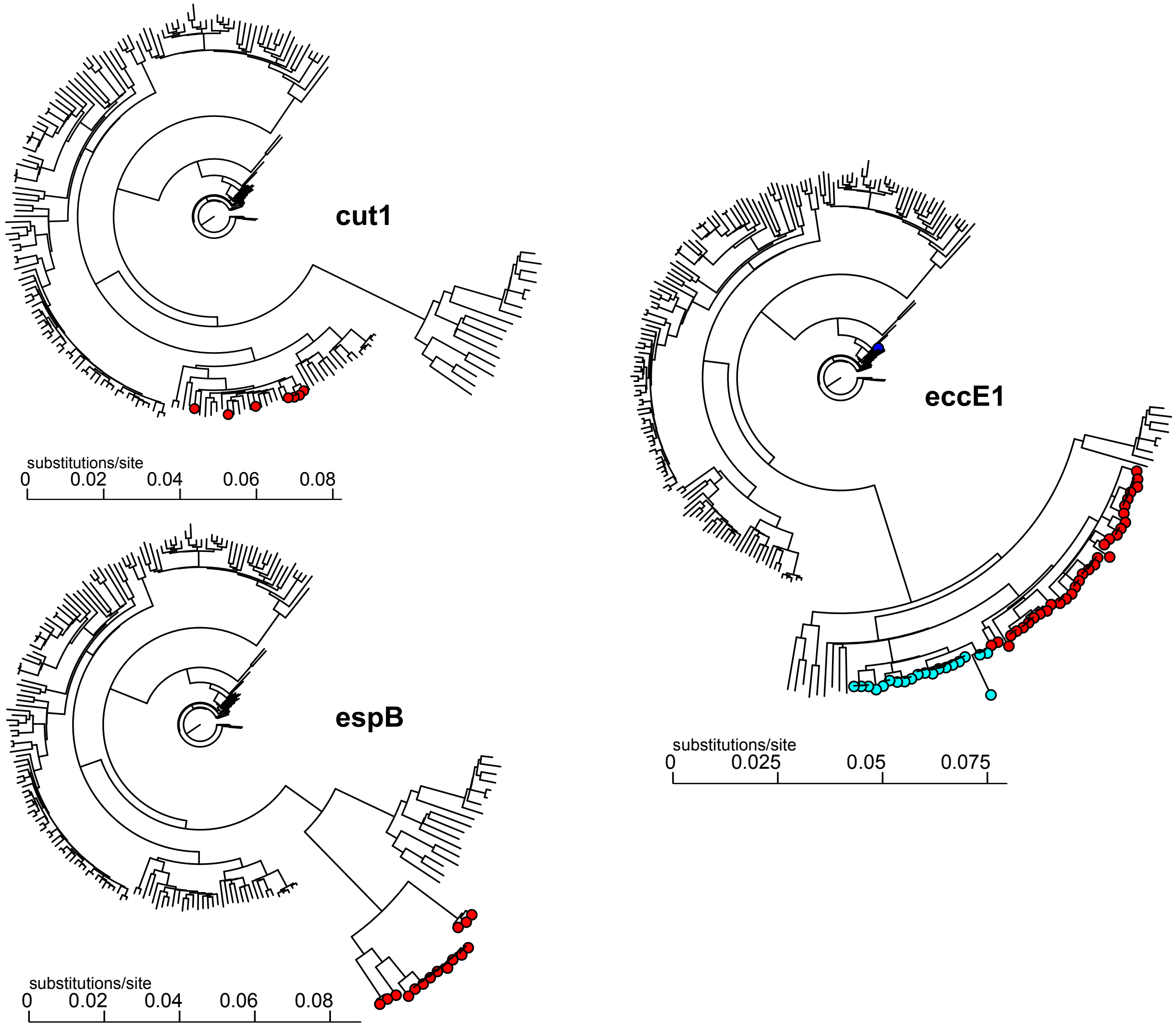

Supplementary figure S7d. Scars restricted to evolutionarily related isolates. Scars from 15 genes were found only in close evolutionary relatives, suggesting that these scars only occurred once in the population analyzed and were derived from a common ancestor. These 15 genes are: *cut1, eccB1, eccCa1, eccD1, espB, fusA2, ltp1, Rv0045c, Rv0176, Rv0458, Rv1132, Rv1575, Rv2216, Rv2561, and Rv0823c*. Each phylogenetic tree depicts one scar (red circles), and the evolutionary relationships between each isolate that contained that scar against a background of 200 non-scarred phylogenetically distant isolates (remaining isolates in the tree). Direct descent of these scars was supported by both Neighbor Joining and Bayesian methods. Only three representative trees are shown. Although unrooted, the Newick tree visualizations are implicitly rooted at the top node.

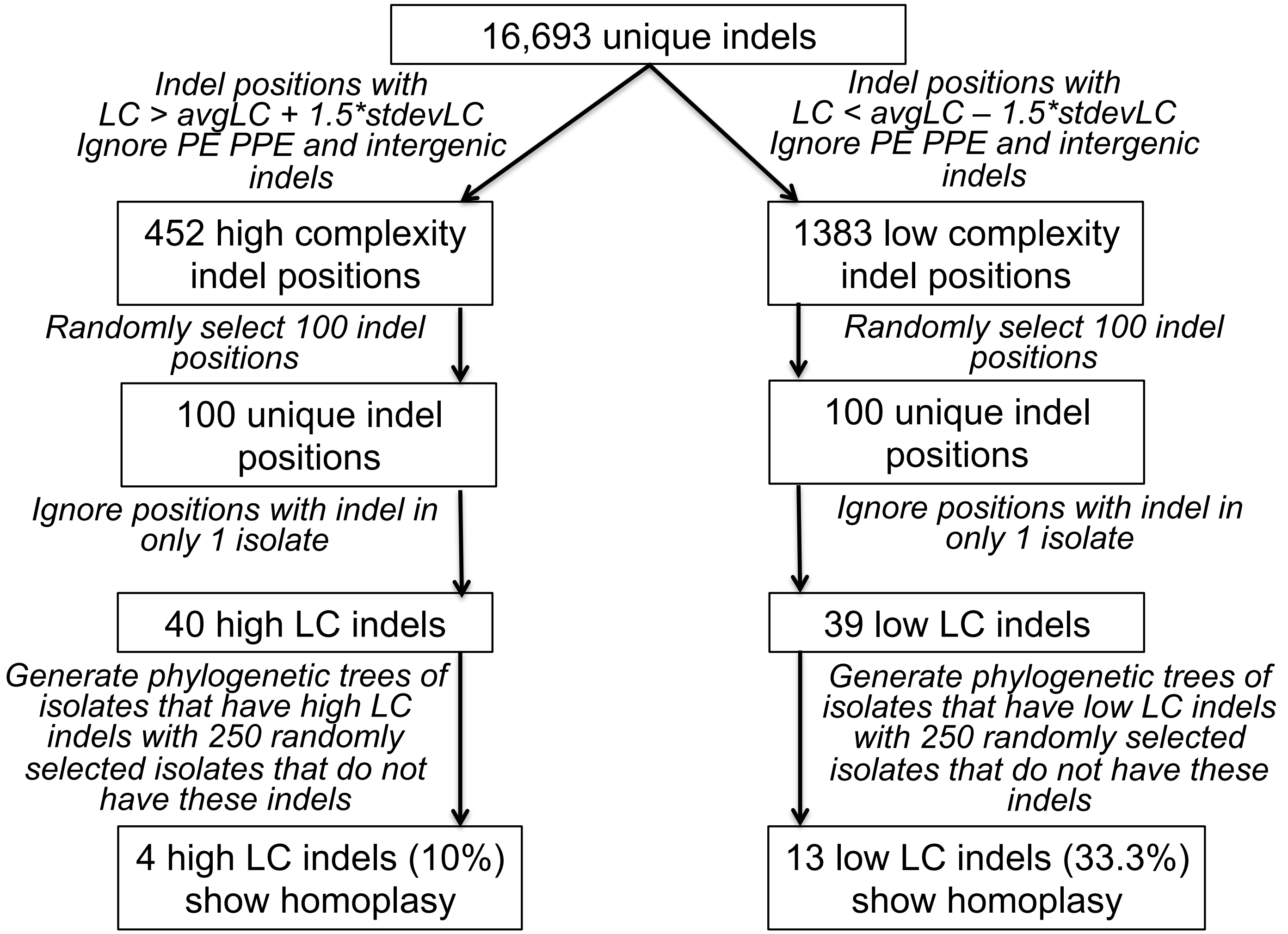

Supplementary figure S8a. Homoplasies in indels in high and low complexity regions. The approach to identity homoplasies in low and high complexity regions is shown. The average genomic *LC* (linguistic complexity) score for the reference *M. tuberculosis* genome is 0.588 with a standard deviation of 0.168. Unique indel positions with extreme complexity scores (1.5 standard deviations away from the mean) were selected. Phylogenetic trees (using Bayesian and Neighbor Joining methods) were constructed for each high/low complexity region indel that occurred in at least 2 isolates (to allow detection of homoplasy) with 250 randomly selected isolates. Visual inspection of the trees showed that indels in low complexity regions were three times more likely to show homoplasy than indels found in high complexity regions. The phylogenetic tree of the four high complexity indels that showed homoplasy is shown in panel b.

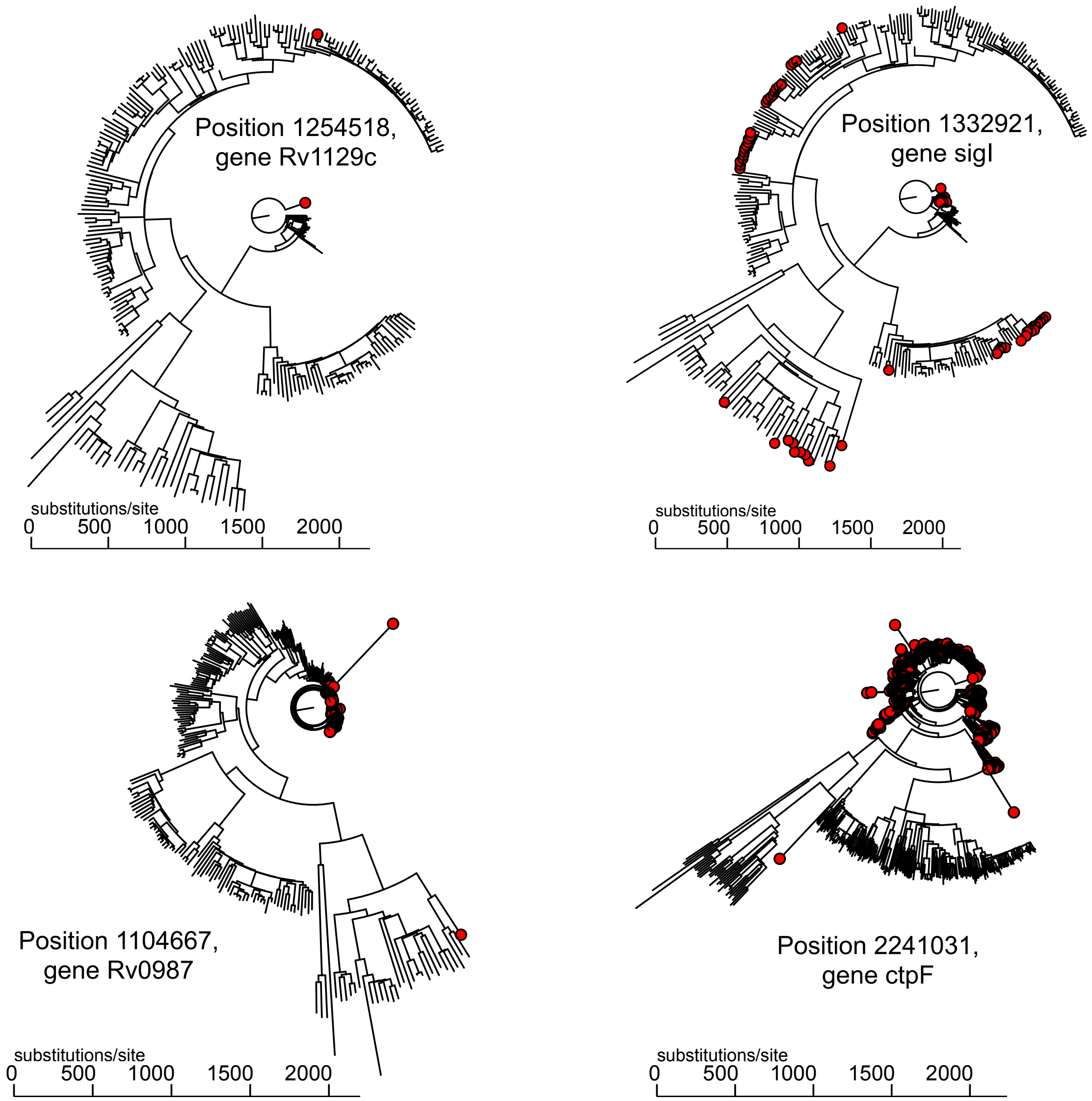

Supplementary figure S8b (each red circle denotes the isolate that had the high complexity indel). The genomic position of indel, and the associated gene name are also specified. Both Bayesian and Neighbor Joining (NJ) phylogenies found homoplasy in high complexity indels at positions 1254518, 1332921, and 1104667. The MCMC simulations for indel at position 2241031 failed to converge after 500,000 generations, thus, homoplasy at this position is confirmed by NJ analysis only. NJ trees are shown for all high complexity indels. Although unrooted, the Newick tree visualizations are implicitly rooted at the top node.

```
N = 6509 (total number of isolates)
initialize all_indels_set to {}
for each isolate:
      initialize isolate_indel_set to {}
      initialize isolate_indel_genes to {}
      if mapping rate < 50% and avg genome-wide coverage < 20:
            ignore isolate
      else:
            for each genomic site in the isolate mpileup file:
                  if coverage > 20 and avg. mapping quality > 10:
                        if detect indel:
                              if indel is present in at least half the reads
                              and forward/reverse balance >= 0.05:
                                    save indel in isolate_indel_set
                                    save indel in all_indels_set
      for each indel in indel_set:
            if indel present in a gene:
                  save gene name in isolate_indel_genes
      for each gene in isolate_indel_genes:
            if > 1 indels present in the gene:
                  if only in-frame indels present:
                        ignore
                  else if at least one frame-shift indel present:
                        introduce all indels simultaneously in the reference gene sequence
                        compare the translated gene product of mutated gene sequence to the
                        reference protein sequence
                        if the translated gene product has stop codon or garbled sequence
                        from indel site(s) to end of protein:
                              ignore this set of indels
                        else if translated gene product matches reference protein sequence
                        from both ends:
                              report the set of indels in this gene as "scar indels"
                              report the gene as scarred gene

analyze unique indels in the all_indels_set for properties and distribution of indels in
the genome.
```

Figure S9. Pseudocode of the ScarTrek program.

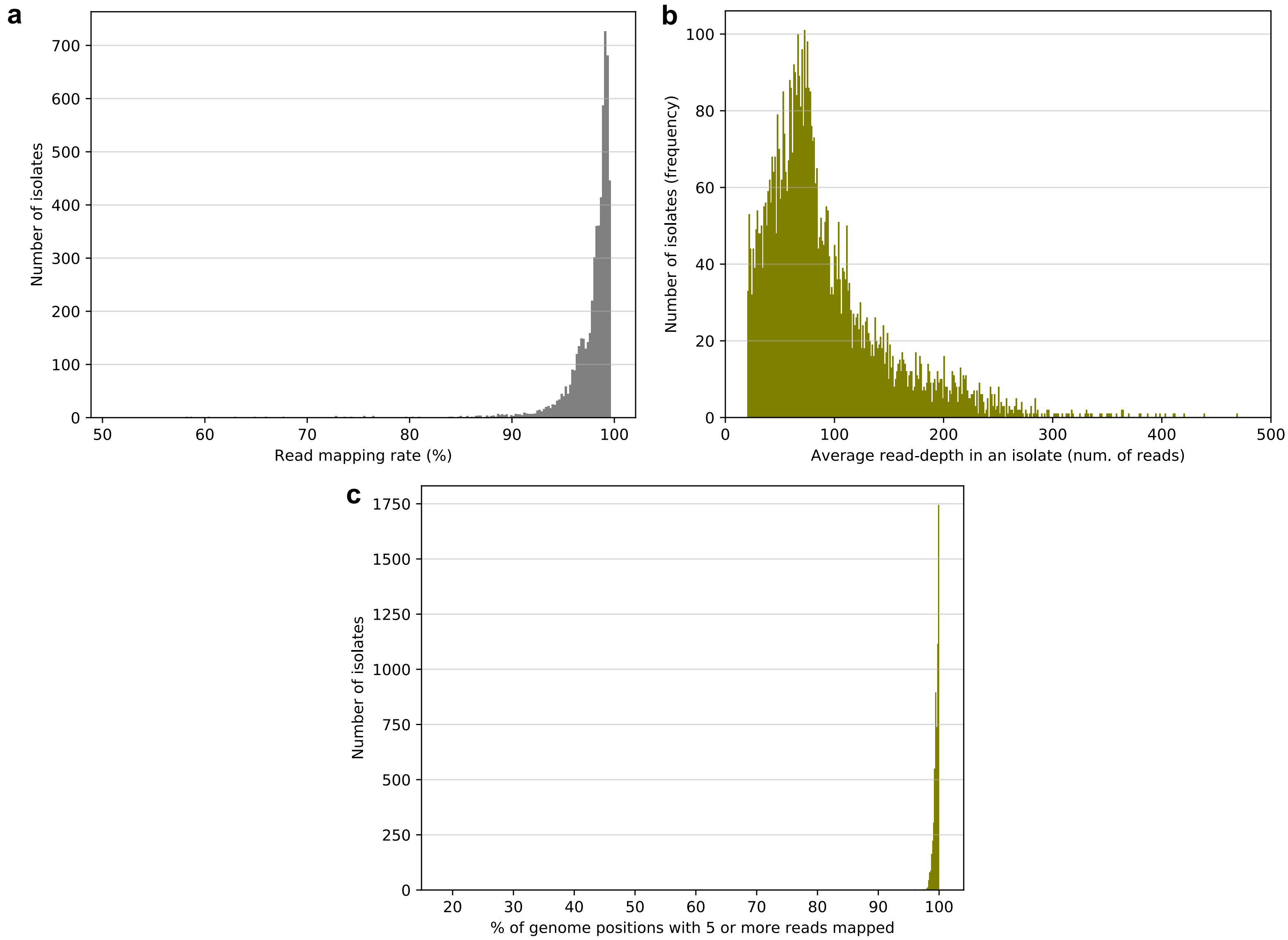

Supplementary figure S10. Data quality. Read mapping percentage (panel a) and average genome-wide coverage (number of reads mapped at each position in the genome on average, panel b) is shown for the 5,977 isolates in the dataset. The majority of samples had >95% reads mapped to the reference genome (strain H37Rv). Isolates with average genome-wide coverage < 20 were excluded from the analyses. Panel (c) shows the percent of genomic sites that were mapped by 5 or more reads (all isolates had at least 95% of the reference genome mapped by reads).

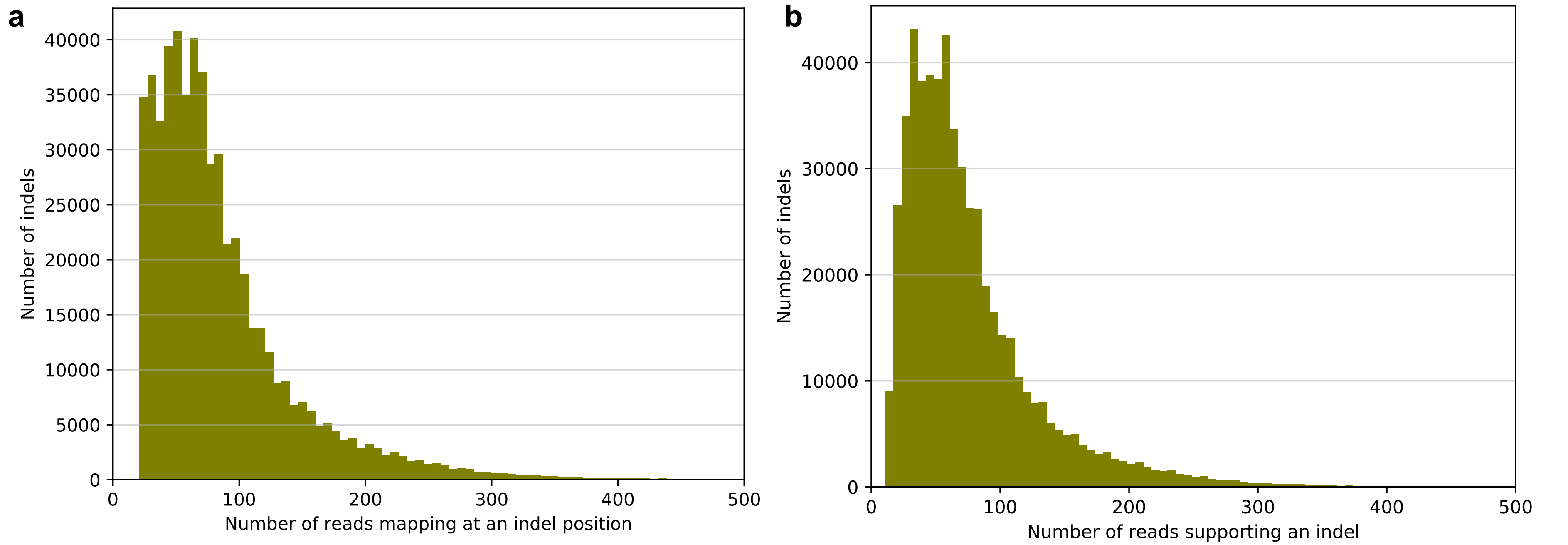

Supplementary figure S11. Quality of read mapping at all indel positions (See Supplementary Dataset 5 for data plotted in this figure). (a) The read depth is shown for each indel call in the 5,977 isolates (each indel position had a minimum depth of 20X). (b) The number of reads that support an indel call are shown for each indel (each indel had a minimum support of 10 reads).

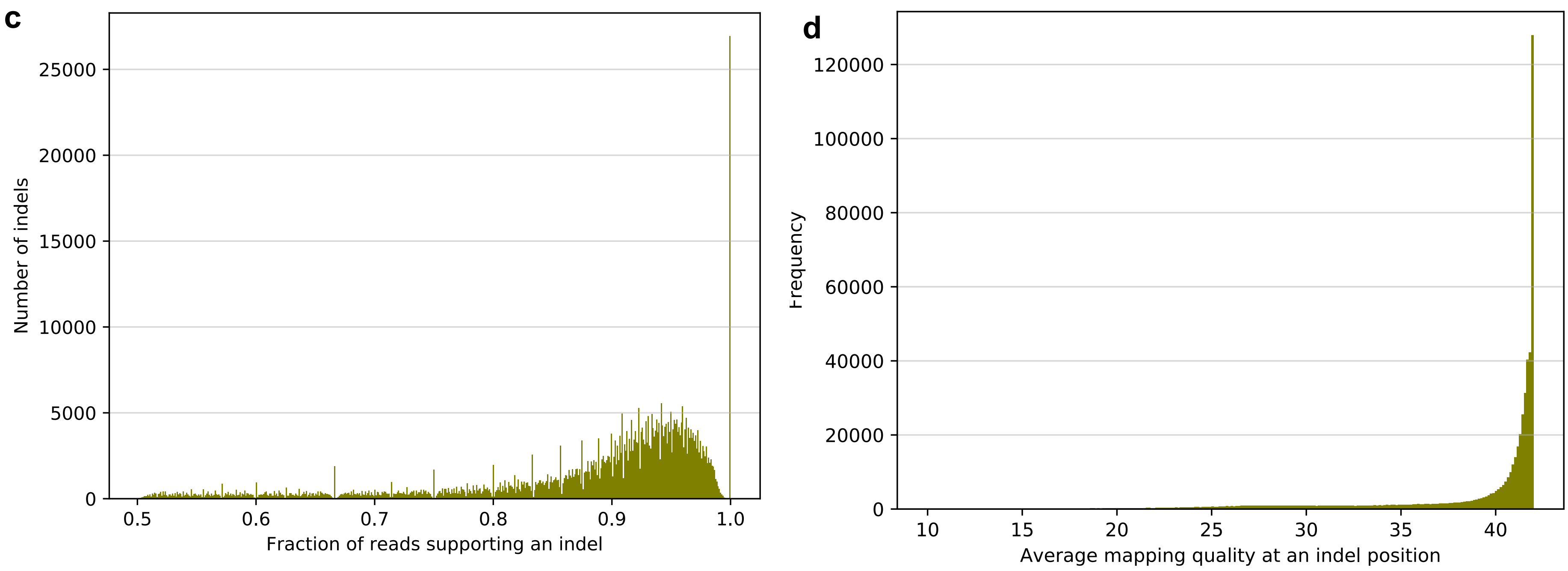

Supplementary figure S11 contd. (c) The fraction of reads that support an indel call are shown for each indel. (d) The average mapping quality is shown for each indel call. Indels with average mapping quality <= 10 are excluded from the study.

e

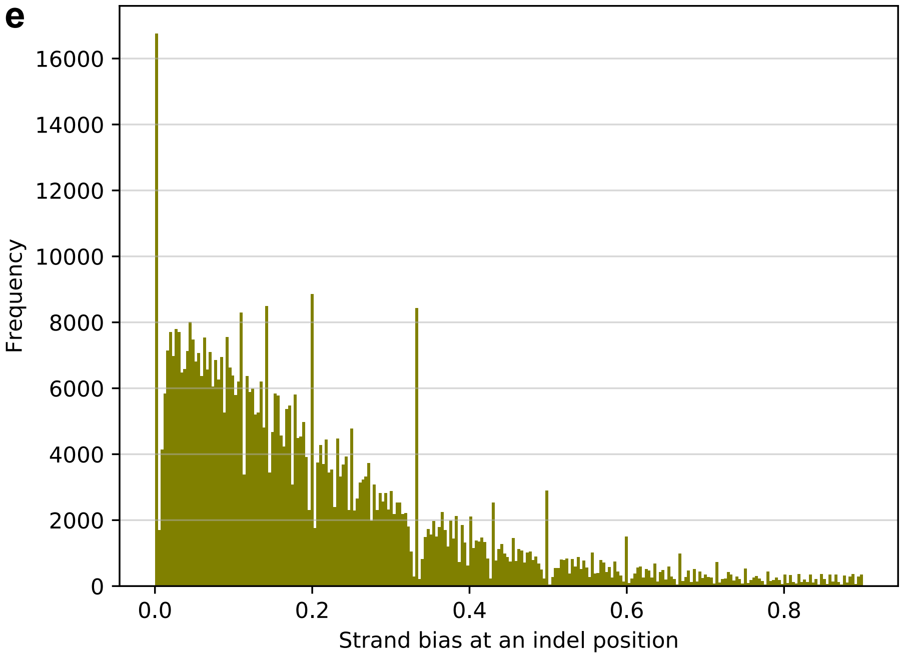

Supplementary figure S11 contd. (e) The strand bias at each indel call is shown. Strand bias here is defined as *sb = abs(numf – numr)/(numf + numr)*, where *numf* and *numr* are the number of forward and reverse reads supporting an indel. Thus, if *numf* = *numr* (i.e., equal support on forward and reverse reads), *sb = 0*. If all of the read support comes from either the forward reads (*numr* = 0) or the reverse reads (*numf* = 0), then *sb* = 1. Thus *sb* ranges from [0,1]. A value of *sb* closer to 1 denotes strand bias, and indels with high strand bias (or low forward/reverse balance, see methods) are not included in the analysis.

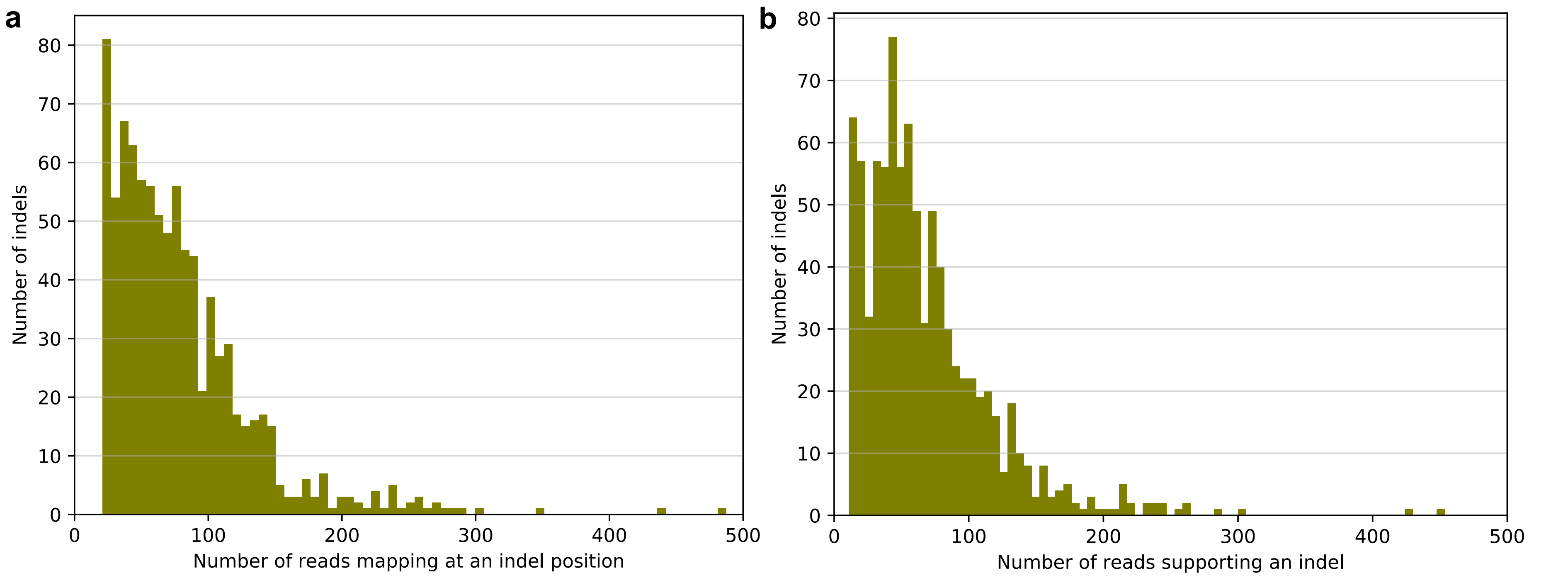

Supplementary figure S12. Quality of read mapping at scar indel positions. (a) The read depth is shown for each scar indel call (each scar indel position had a minimum depth of 20X). (b) The number of reads that support the indel call are shown for each scar indel (each scar indel had a minimum support of 10 reads).

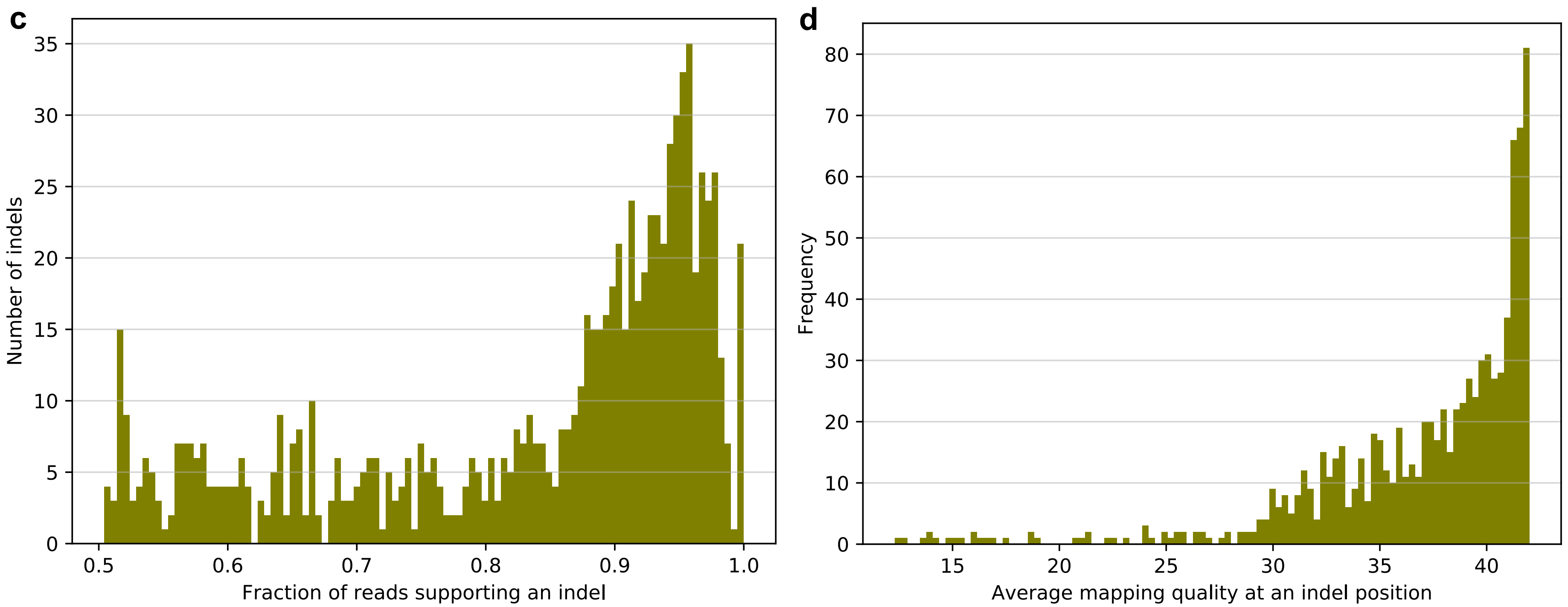

Supplementary figure S12 contd. (c) The fraction of reads that support an indel call are shown for each scar indel. (d) The average mapping quality is shown for each scar indel call. Scar indels with average mapping quality <= 10 are excluded from the study.

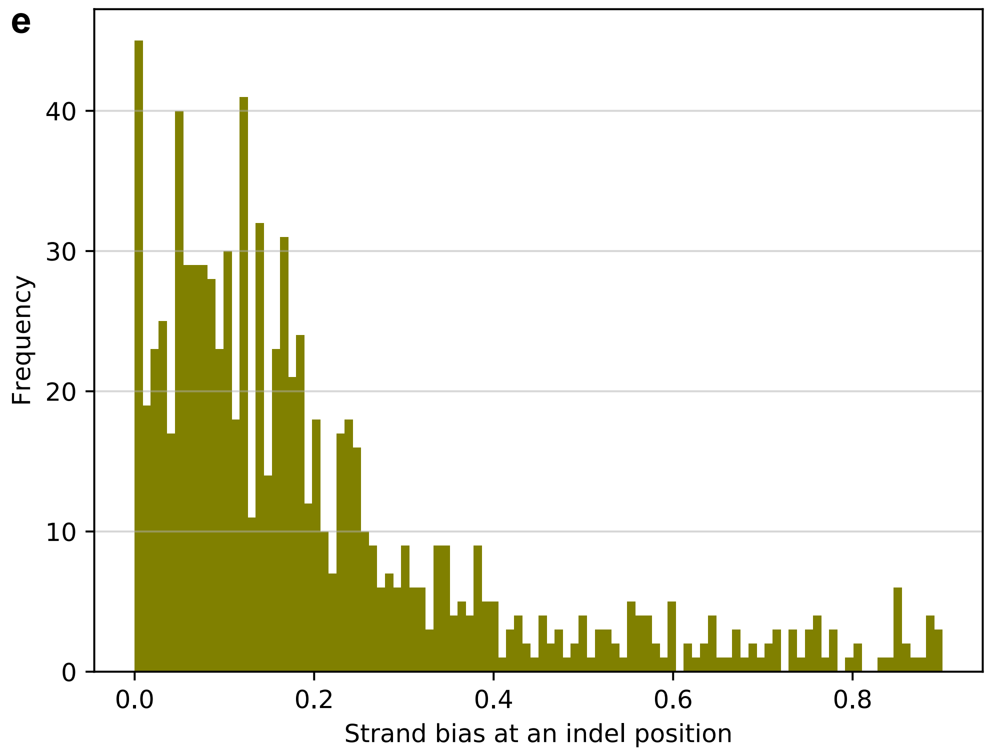

Supplementary figure S12 contd. (e) The strand bias at each scar indel call is shown. Strand bias here is defined as *sb* = *abs(numf – numr)/(numf* + *numr)*, where *numf* and *numr* are the number of forward and reverse reads supporting an indel. Thus, if *numf* = *numr* (i.e., equal support on forward and reverse reads)*, sb = 0*. If all of the read support comes from either the forward reads (*numr* = 0) or the reverse reads (*numf* = 0), then *sb* = 1. Thus *sb* ranges from [0,1]. A value of *sb* closer to 1 denotes strand bias, and indels with high strand bias (or low forward/reverse balance, see methods) are not included in the analysis.

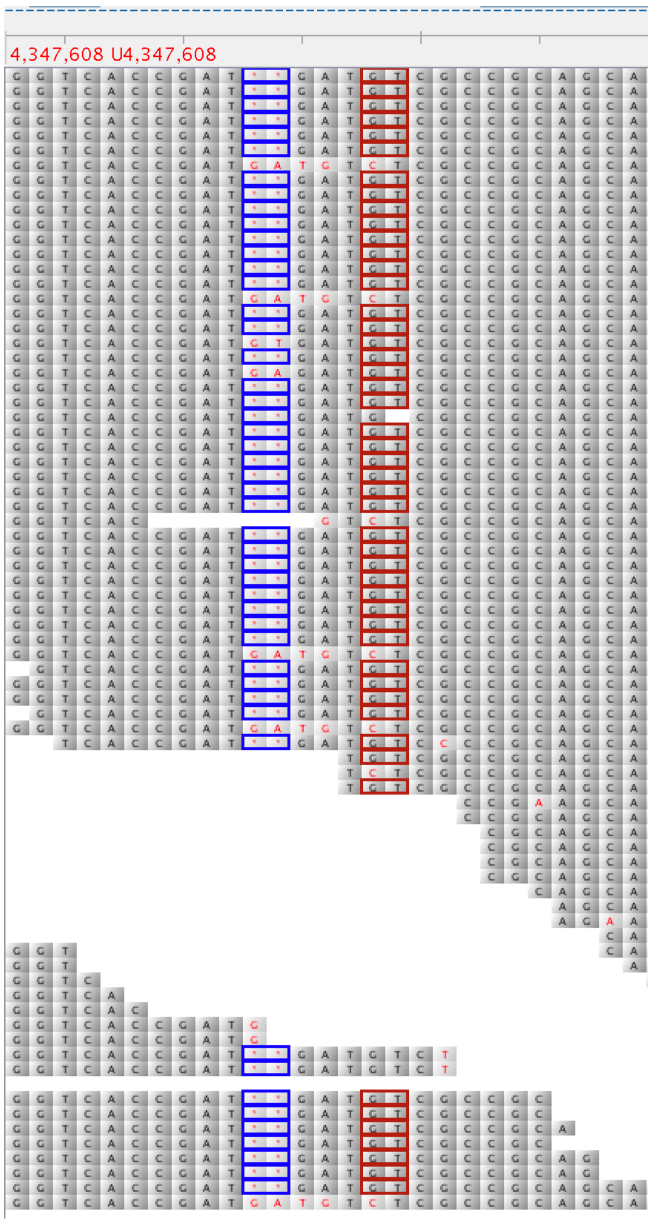

Supplementary figure S13. a) Snapshot of reads mapped to indels of a scar in the gene *eccCa1*. The scar indels are T_4347617_-2CG and G_4347623_+2TC. Deletions are in blue and insertions are in red. The read alignments are viewed by Tablet. See panels b and c for examples of scars that were rejected upon manual inspection.

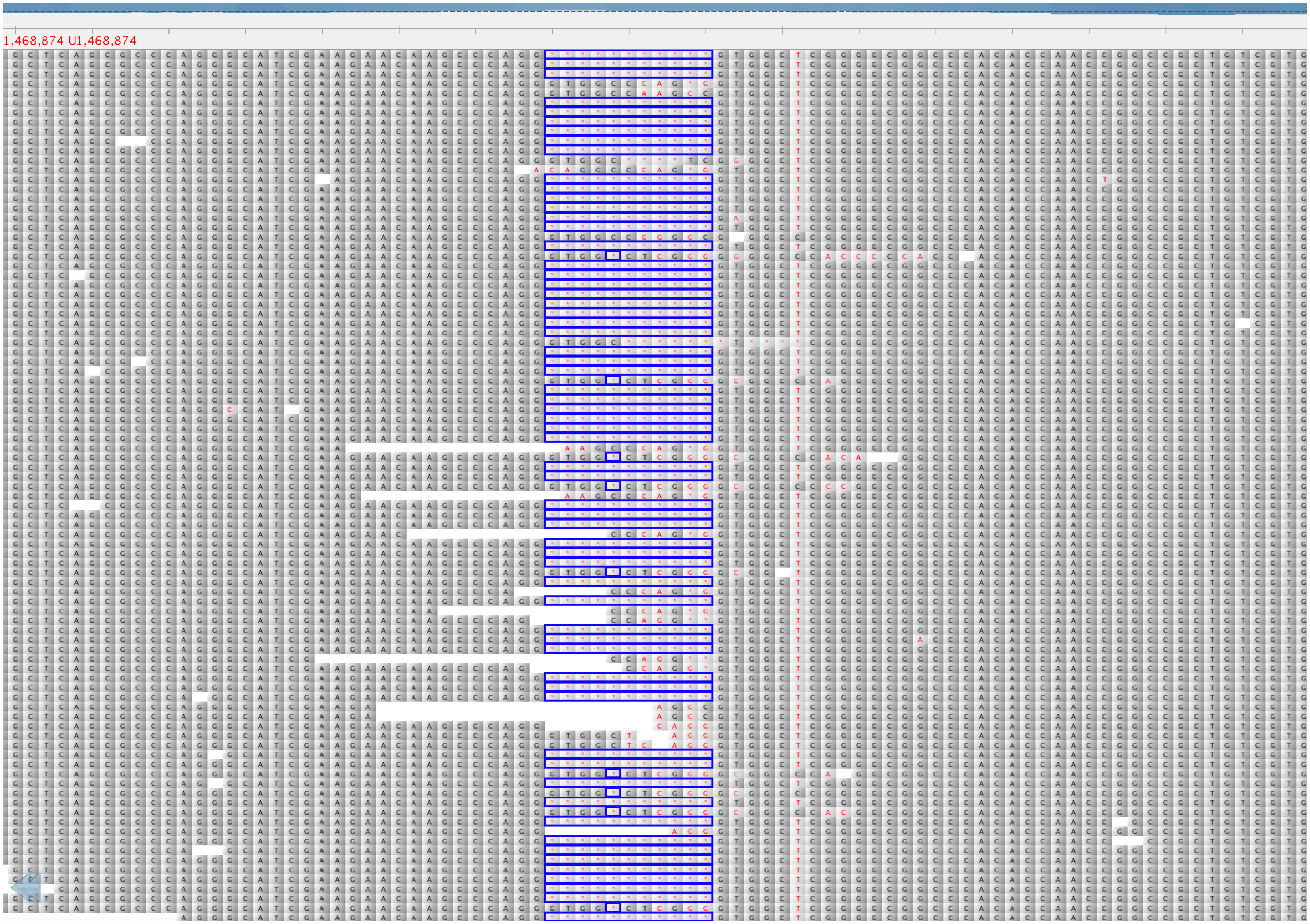

Supplementary figure S13b). Reads aligned to indels in a putative scar identified by ScarTrek in gene *Rv1313c* that was rejected upon visual inspection of mapped reads. The indels G_1468909_-11GTGGCCTTGTC and G_1468913_-1C were present exclusively in different reads and did not appear together in a mapped read.

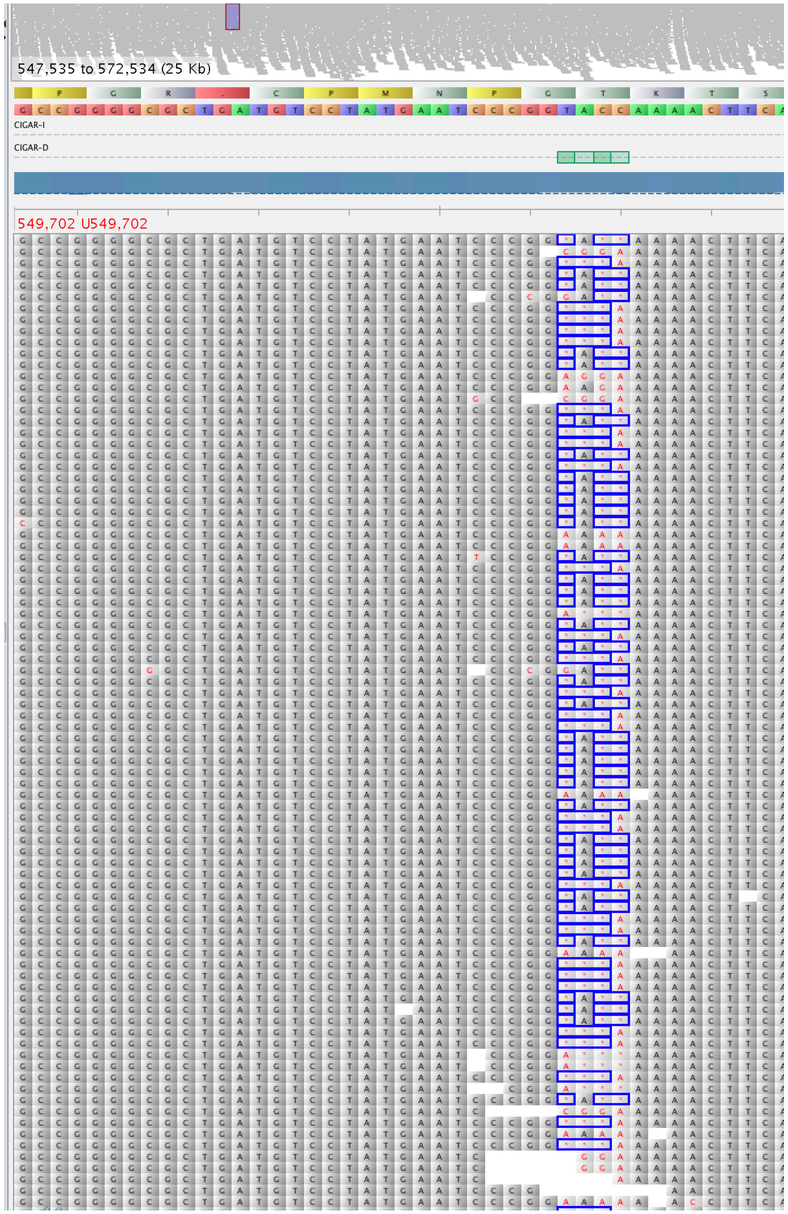

Supplementary figure S13c). Reads aligned to indels in a putative scar identified by ScarTrek in gene *Rv0458* that was rejected upon visual inspection of mapped reads. The two indels (G_549731_-1T and A_549733_-2CC) were interpreted as a single indel of length 3 in some reads, creating confusion regarding the identity of true indels at this site.

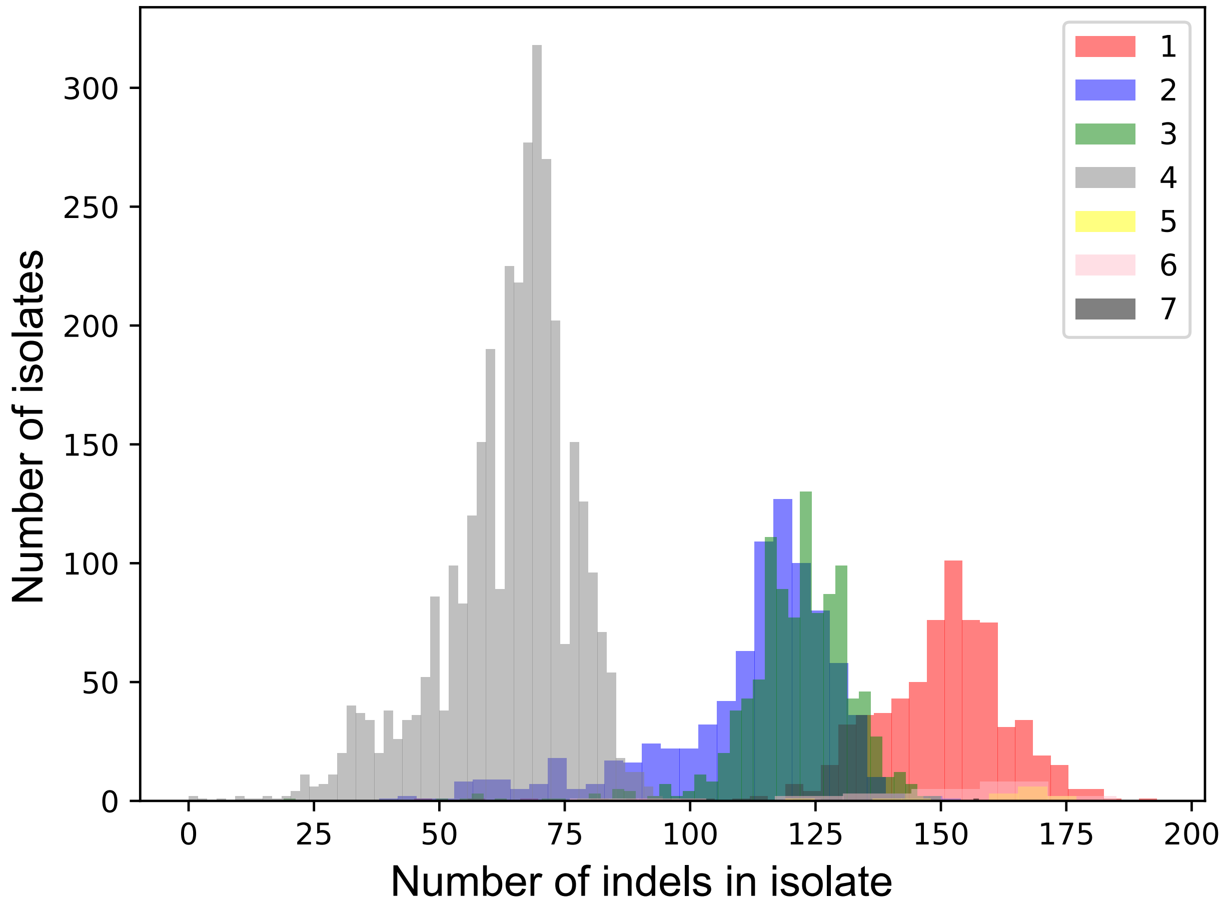

Supplementary figure S14. Histogram of indel rates (number of indels/isolate) in *M. tuberculosis* lineages in our dataset. The *M. tuberculosis* lineage (there are 7 lineages, 1-7) for each isolate in our dataset was determined using the program SNPIT[1]. The indel rate (x-axis) was overlapping for lineages 2 (purple) and 3 (green), two closely related lineages with a most recent common ancestor[2], but were distinct from other M. tuberculosis lineages. Lineages 1 and 5, which show the largest number of indels represent so called "ancient" lineages in contrast to the "modern" lineages 2, 3, and 4, which all show smaller numbers of indels.

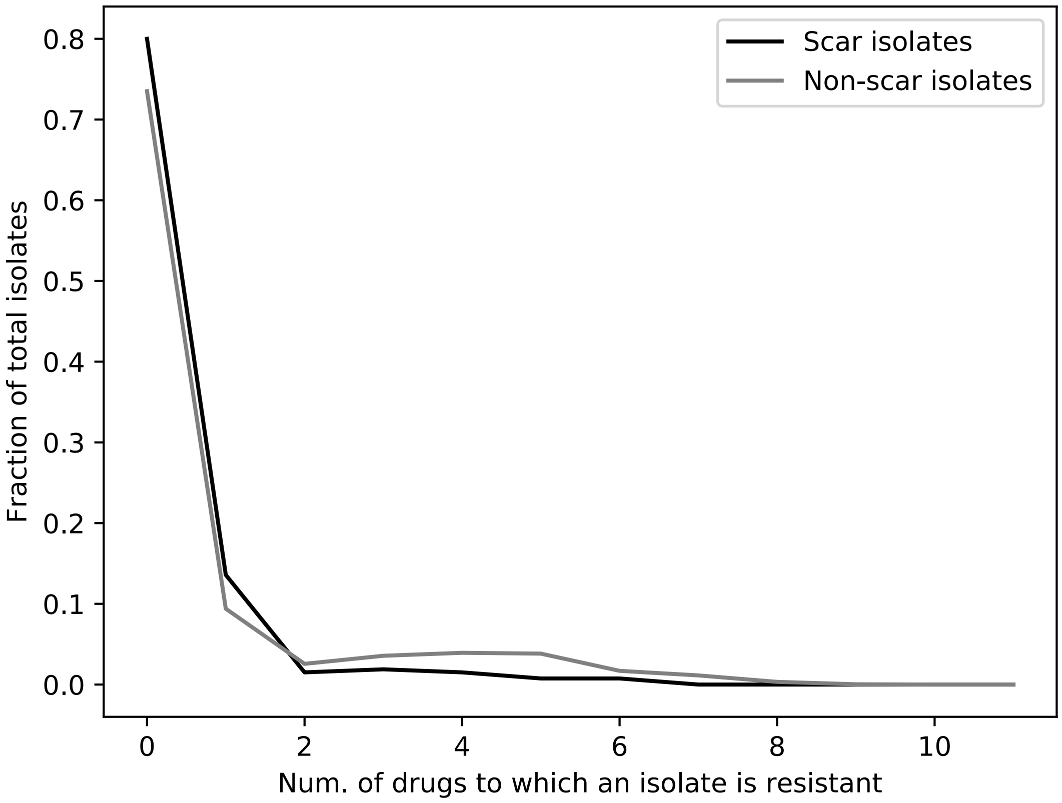

Supplementary figure S15. Drug resistance profiles of the scar and non-scar isolates. We analyzed the phenotypic drug-resistance profiles of the 265 scar isolates and 3001 non-scar isolates from Zhang *et al.*, 2013, Nature Genetics[3] (PMID:23995137) and Walker *et al.*, 2015, Lancet Infectious Diseases[4] (PMID:26116186). The fraction of isolates that are resistant to different number of drugs tested was indistinguishable between the scar and the non-scar isolates.

Supplementary Tables

Supplementary Table S1. Performance of SAMtools, GATK HaplotypeCaller, and ScarTrek on reads simulated from *M. tuberculosis* reference H37Rv. 157 manually confirmed indels were introduced in 10 copies of H37Rv genomes. The reads simulated from these 10 genomes (at three average depths) were then processed as the clinical TB isolates for indel calling. The results of indel calling are reported.

Method	Average depth	No. indels predicted (No. of true indels=157)	No. indels correctly predicted (TP)	No. true indels missed (FN)	No. incorrect indel calls (FP)	Sensitivity TP/(TP+FN)	Positive Predictive Value TP/(TP+FP)
SAMtools	50X	158	93	64	65	0.592	0.589
	100X	205	102	55	103	0.65	0.498
	200X	243	106	51	137	0.675	0.436
GATK HaplotypeCaller	50X	146	101	56	45	0.643	0.692
	100X	152	106	51	46	0.675	0.697
	200X	162	117	40	45	0.745	0.722
ScarTrek	50X	149	112	45	37	0.713	0.752
	100X	155	114	43	41	0.726	0.735
	200X	162	115	42	47	0.732	0.71

Supplementary Table S2. Frequency of unique indels in essential, repeat genes (PE/PPE), and remaining genes in our dataset of 5,977 *M. tuberculosis* clinical isolates.

Num. indels	Num. essential genes	Num. PE/PPE genes	Num. remaining genes (non-essential non PE/PPE)
0	329	7	865
1	96	6	586
2	22	11	467
3	8	11	394
4	1	11	274
5	0	8	216
6	2	6	152
7	1	9	142
8	0	7	91
9	0	4	71
10	0	8	47
>10	0	77	182

Supplementary Table S3. Microbial genomes from GenBank matching the WGS data from 9 isolates that had indels in the 16S ribosomal (rrs) gene. Top hits from comparing the k-mer signatures (k=31) derived from raw reads of the isolates with *rrs* indels (queries) to the 100k GenBank Microbial Genomes database using 'gather' command of package 'sourmash' is shown. A 2nd hit is reported if more than one hit match the query with >10% of its kmers. Contig/scaffold/genome length of the hits are shown.

SRA ID of isolate (query)	% reads mapping to Mtb (H37Rv)	GenBank ID of top sourmash gather hit	Length of top hit (bp)	% of top hit's kmers that match query	GenBank ID of 2nd sourmash gather hit	Length of 2nd hit (bp)	% of 2nd hit's kmers that match query
ERR1063836	99.18	KK340201.1 Mycobacterium tuberculosis	822452	99.0	-	-	-
ERR126624	99.42	CPFS01000001.1 Mycobacterium tuberculosis	281315	97.4	-	-	-
ERR245649	99.44	MMMR01000001.1 Mycobacterium tuberculosis	3121967	98.6	-	-	-
ERR046834	99.62	CNCS01000001.1 Mycobacterium tuberculosis	178491	97.4	-	-	-
SRR2100157	99.04	MMCG01000001.1 Mycobacterium tuberculosis	844019	97.4	-	-	-
SRR2100249	93.5	KL406390.1 Mycobacterium tuberculosis	377725	98.4	-	-	-
SRR2100473	97.94	KK340113.1 Mycobacterium tuberculosis	463872	99.5	-	-	-
SRR2100695	95.52	CP009177.1 Mycobacterium tuberculosis	4411532	99.3	-	-	-
SRR2100959	96.51	KK357896.1 Mycobacterium tuberculosis	4084878	98.1	-	-	-

Supplementary Table S4: Essential genes with a frame-shifting indel in the first gene segment (within the first 10% of the gene sequence).

Gene	Gene start	Gene end	Complement gene?	Gene length	Indel position in gene	Distance from indel to nearest alternative ATG start codon (nt)	Genic position of 1st ATG start codon	Indel in region preceding 1st ATG start codon?	Gene product
ftsK	3059855	3062506	Yes	2652	103	15	1	No	Possible cell division transmembrane protein
ftsK	3059855	3062506	Yes	2652	18	101	1	No	Possible cell division transmembrane protein
ftsK	3059855	3062506	Yes	2652	10	108	1	No	Possible cell division transmembrane protein
wag31	2404616	2405398	Yes	783	1	25	1	No	Diviva family protein Wag31
espG3	352149	353036	No	888	9	94	1	No	ESX-3 secretion-associated protein
htrA	1365875	1367461	No	1587	26	5	32	Yes	Probable serine protease
Rv2147c	2406118	2406843	Yes	726	57	57	70	Yes	Conserved hypothetical protein
Rv2147c	2406118	2406843	Yes	726	2	2	70	Yes	Conserved hypothetical protein
eccA3	342130	344025	No	1896	66	15	1	No	ESX conserved component
murE	2419001	2420608	Yes	1608	56	556	612	Yes	Probable UDP-N-acetylmuramoylalanyl-D-glutamate-2,6-diaminopimelate ligase
murE	2419001	2420608	Yes	1608	61	551	612	Yes	Probable UDP-N-acetylmuramoylalanyl-D-glutamate-2,6-diaminopimelate ligase
Rv0647c	741151	742617	Yes	1467	67	53	1	No	Conserved protein
guaA	3812501	3814078	Yes	1578	2	74	76	Yes	Probable GMP synthase
aftA	4237932	4239863	No	1932	57	136	1	No	Arabinofuranosyltransferase
Rv1990c	2233296	2233637	Yes	342	1	8	1	No	Probable transcriptional regulatory protein
glgE	1492320	1494425	Yes	2106	42	20	62	Yes	Probable glucanase GlgE
engA	1940288	1941679	No	1392	2	104	106	Yes	Probable GTP-binding

									protein
pabB	1122222	1123598	Yes	1377	59	141	200	Yes	Probable para-aminobenzoate synthase component I PABD
fadD32	4261153	4263066	Yes	1914	17	7	1	No	Fatty-acid-AMP ligase
Rv0810c	904905	905087	Yes	183	1	159	1	No	Conserved hypothetical protein
sucD	1063140	1064051	No	912	5	4	1	No	Probable succinyl-CoA synthetase
Rv3034c	3394019	3394921	Yes	903	45	55	100	Yes	Possible transferase
Rv3034c	3394019	3394921	Yes	903	67	33	100	Yes	Possible transferase
Rv0228	273055	274278	No	1224	26	97	1	No	Probable integral membrane acyltransferase

Supplementary Table S5. Mean *LC* and *H* scores of indel and non-indel positions compared using the Welch t-test. *LC* and *H* scores of the 21 nucleotide-long sequences centered at an indel were compared to 21-nucleotide long sequences not containing an indel. Since there are more than 4.3 million non-indel positions, they are randomly sampled without replacement (10 independent samples) to match the number of indel positions. Non-indel positions within 20 nucleotides of an indel position were excluded in the random samples to ensure that the complexity scores of the non-indel position were not influenced by the complexity of an indel region.

Sample No.	No. indel positions	No. non-indel positions	Mean *LC* score for indel positions	Mean *LC* score for non indel positions	Two-tailed p-value of Welch t-test for comparing indel and non-indel *LC* scores	Mean *H* score for indel positions	Mean *H* score for non indel positions	Two-tailed p-value of Welch t-test for comparing indel and non-indel *LC* scores
1	16693	16385	0.541	0.59	1.45e-133	0.928	0.94	3.37e-118
2	16693	16463	0.541	0.591	2.39e-137	0.928	0.94	2.63e-121
3	16693	16387	0.541	0.589	2.53e-125	0.928	0.94	8.72e-116
4	16693	16377	0.541	0.589	1.06e-128	0.928	0.941	5.6e-123
5	16693	16396	0.541	0.59	1.56e-133	0.928	0.94	1.43e-117
6	16693	16371	0.541	0.59	1.71e-134	0.928	0.94	2.66e-112
7	16693	16494	0.541	0.591	8.53e-137	0.928	0.94	6.4e-118
8	16693	16495	0.541	0.592	6.05e-148	0.928	0.941	1.92e-129
9	16693	16397	0.541	0.59	6.68e-133	0.928	0.941	4.37e-123
10	16693	16463	0.541	0.591	1.26e-138	0.928	0.941	4.42e-128

Supplementary Table S6. The set of indels that cause and then restore a frameshift (thus creating a "scar") in the clinical isolates of *Mycobacterium tuberculosis* are listed. Genic nucleotide positions from the translational start site and indel nucleotides are shown for each scar according to the *M. tuberculosis* H37Rv reference genome (Genbank ID: AL123456.3). Genome coordinates of each indel are in parenthesis. '+' sign indicates insertions and '-' sign denotes deletions. Scar indel sets were sometimes found multiple times in the same study or across studies (last three columns).

Gene #	Scar #	Gene	Scar indel set (frameshift introducing and restoring indels)	# in Zhang *et al.*, 2013	# in Guerra-Assunção *et al.*, 2015	# in Walker *et al.*, 2015
1	1	Rv0176	- 864 (208316) ['T']; + 869 (208321) ['C']	0	32	36
2	2	cyp128	- 707 (2543569) ['T']; + 703 (2543573) ['CCGA']	0	1	0
3	3	espI	- 452 (4353462) ['C']; + 591 (4353601) ['GCGA']	0	1	0
	4	espI	- 209 (4353219) ['C']; + 347 (4353357) ['C']	0	3	0
	5	espI	- 452 (4353462) ['C']; + 498 (4353508) ['A']	0	0	1
	6	espI	+ 1067 (4354077) ['C']; - 1277 (4354287) ['G']	0	0	3
	7	espI	+ 296 (4353306) ['C']; - 452 (4353462) ['C']	0	0	2
	8	espI	+ 1320 (4354330) ['TGGCTCA']; - 1338 (4354348) ['A']	0	0	2
	9	espI	- 1394 (4354404) ['G']; + 1452 (4354462) ['A']	0	0	42
	10	espI	+ 296 (4353306) ['C']; - 654 (4353664) ['C']	0	0	1
	11	espI	- 149 (4353159) ['C']; + 452 (4353462) ['CC']; - 945 (4353955) ['C']; - 1394 (4354404) ['G']; + 1452 (4354462) ['A']	0	0	1
	12	espI	- 627 (4353637) ['C']; + 654 (4353664) ['C']; - 1394 (4354404) ['G']; + 1452 (4354462) ['A']	0	0	1
4	13	Rv2542	+ 1094 (2866224) ['G']; - 1098 (2866228) ['A']	0	4	2
5	14	espB	- 863 (4361062) ['C']; + 840 (4361085) ['C']	0	5	10
6	15	sigM	+ 216 (4400402) ['A']; - 474 (4400660) ['C']	1	1	0
	16	sigM	+ 167 (4400353) ['A']; - 474 (4400660) ['C']	0	0	1
7	17	eccE1	+ 335 (4363085) ['T']; - 331 (4363089) ['C']	0	22	11
	18	eccE1	- 106 (4363314) ['G']; - 65 (4363355) ['CC']	0	19	1
	19	eccE1	- 224 (4363196) ['C']; + 201 (4363219) ['G']	0	0	1
8	20	pks12	- 712 (2306274) ['G']; + 707 (2306279) ['C']	0	9	9
	21	pks12	+ 6781 (2300205) ['G']; - 6777 (2300209) ['C']	1	35	37
9	22	eccCa1	- 1136 (4347617) ['CG']; + 1142 (4347623) ['TC']	0	1	0
	23	eccCa1	- 745 (4347226) ['C']; + 759 (4347240) ['T']	0	0	2
	24	eccCa1	- 518 (4346999) ['A']; - 566 (4347047) ['CC']	0	0	1
	25	eccCa1	- 1072 (4347553) ['G']; + 1095 (4347576) ['T']	0	0	2
	26	eccCa1	+ 634 (4347115) ['C']; - 641 (4347122) ['A']	0	0	4
10	27	Rv0823c	- 961 (916685) ['C']; - 958 (916688) ['GC']	0	0	9

11	28	Rv0458	- 56 (549731) ['T'] - 58 (549733) ['CC'];	0	2	17
12	29	Rv0045c	- 574 (49365) ['C'] - 249 (49690) ['GG']	0	5	0
	30	Rv0045c	+ 814 (49125) ['GTGTGGGT']; + 811 (49128) ['AC']; + 809 (49130) ['T']; + 805 (49134) ['CGCCATC'];	0	0	1
13	31	fadE35	+ 1700 (4252785) ['T']; + 1702 (4252787) ['CT']	0	0	1
14	32	Rv0397	- 145 (475961) ['T']; + 148 (475964) ['C']	0	0	1
15	33	Rv0393	+ 1114 (473895) ['T']; - 1116 (473897) ['C']	0	0	1
16	34	ptrBa	+ 601 (874834) ['GC']; + 602 (874835) ['G']	0	0	1
17	35	aofH	+ 625 (3539130) ['C']; - 634 (3539139) ['G']	0	0	5
	36	aofH	+ 618 (3539123) ['G']; - 634 (3539139) ['G']	0	0	2
18	37	mycP2	- 557 (4369613) ['C']; - 434 (4369736) ['ATTGCCGCTGCACC']	0	0	1
19	38	glpQ1	- 571 (4314991) ['C'] + 325 (4315237) ['G']	0	0	1
20	39	Rv2561	+ 180 (2881589) ['G']; - 188 (2881597) ['G']	0	0	1
	40	Rv2561	- 188 (2881597) ['G']; + 199 (2881608) ['C']	0	0	1
	41	Rv2561	+ 49 (2881458) ['A']; - 188 (2881597) ['G']	0	0	2
	42	Rv2561	- 188 (2881597) ['G']; + 267 (2881676) ['A']	0	0	1
	43	Rv2561	+ 173 (2881582) ['T']; - 188 (2881597) ['G']	0	0	1
21	44	sppA	- 6 (815669) ['T']; + 17 (815680) ['A']	0	0	21
22	45	Rv0470A	- 232 (562062) ['TGGC']; + 192 (562102) ['A']	0	0	1
23	46	lipF	- 642 (3906365) ['G']; + 545 (3906462) ['A']	0	0	1
24	47	ctpI	+ 4711 (125830) ['T']; - 4658 (125883) ['G']	0	0	1
25	48	eccD1	+ 33 (4355040) ['CC']; + 37 (4355044) ['G']	0	0	2
	49	eccD1	- 846 (4355853) ['G']; + 856 (4355863) ['G']	0	0	1
	50	eccD1	+ 846 (4355853) ['G']; - 856 (4355863) ['G']	1	0	0
26	51	fusA2	+ 161 (147610) ['GTGCC']; + 159 (147612) ['T']	0	0	2
27	52	ptrBb	+ 102 (874834) ['GC']; + 103 (874835) ['G']	0	0	1
28	53	ctpC	+ 1507 (3652033) ['G']; - 1521 (3652047) ['C']	0	0	1
29	54	esxA	- 91 (4352700) ['G']; + 101 (4352710) ['T']	0	0	1
30	55	Rv3094c	- 321 (3463570) ['G']; + 265 (3463626) ['G']	0	0	1
31	56	Rv0249c	+ 48 (301607) ['C']; - 41 (301614) ['T']	0	0	1
32	57	Rv1132	- 492 (1257817) ['AT']; - 500 (1257825) ['G']	0	0	7
33	58	Rv2885c	+ 1307 (3194241) ['C']; - 1194 (3194354) ['CCAACGT']	0	0	1
34	59	Rv1264	- 485 (1412379) ['A']; + 489 (1412383) ['C']	0	0	1
35	60	pknH	+ 1053 (1414787) ['G']; + 899 (1414941) ['GG']	0	0	1
36	61	cut1	+ 17 (1989059) ['C']; + 19 (1989061) ['CC'];	0	0	5
37	62	Rv3725	+ 750 (4170964) ['A']; - 917 (4171131) ['A']	0	0	1
38	63	Rv1179c	- 2250 (1311049) ['C']; - 1940 (1311359) ['GATAC']	0	0	1

39	64	Rv2262c	- 1028 (2534524) ['C']; - 990 (2534562) ['TC']	0	0	1
40	65	fadD29	+ 143 (3302312) ['AT']; - 131 (3302324) ['CTCGC'];	0	0	1
41	66	Rv3785	+ 558 (4231878) ['ACAA']; - 648 (4231968) ['A']	0	0	1
42	67	Rv2216	- 505 (2484131) ['G']; + 516 (2484142) ['A']	0	0	2
43	68	ethA	+ 1137 (4326336) ['G']; + 1135 (4326338) ['CG']	0	0	1
44	69	fadD11	+ 300 (1754016) ['C']; - 663 (1754379) ['G']	0	0	1
45	70	eccB1	+ 326 (4345365) ['A']; - 338 (4345377) ['G']	0	0	1
	71	eccB1	+ 307 (4345346) ['G']; - 313 (4345352) ['G']	0	0	3
46	72	Rv1575	- 261 (1780460) ['C']; + 387 (1780586) ['G']	0	0	7
47	73	ltp1	+ 18 (3100151) ['G']; - 15 (3100154) ['G']	0	0	6
48	74	vapB18	- 97 (2867880) ['A']; - 171 (2867954) ['CAACG']	1	0	0

Supplementary Table S7. Frequency of scars and scar indels, and scar conversion rate. The number of isolates that have a scarred gene (m) and number of isolates that have at least one of the scar indels in the gene (n) are shown. Note that some isolates had scars or scar indels in multiple genes, and are counted once for each scarred gene or scar indel they have. Genes in the ESX-1 gene-cluster are highlighted in bold.

**Gene #**	**Gene**	**# isolates with scar in this gene (m)**	**# isolates with at least one scar indel from the gene (n)**
1	pks12	91	115
2	Rv0176	68	736
**3**	**espI**	**57**	**63**
**4**	**eccE1**	**54**	**54**
5	sppA	21	50
6	Rv0458	19	51
**7**	**espB**	**15**	**16**
**8**	**eccCa1**	**10**	**10**
9	Rv0823c	9	45
10	Rv1575	7	2779
11	aofH	7	671
12	Rv1132	7	123
13	Rv2561	6	5776
14	Rv0045c	6	1824
15	Rv2542	6	17
16	ltp1	6	7
17	cut1	5	8
**18**	**eccD1**	**4**	**7**
**19**	**eccB1**	**4**	**4**
20	sigM	3	5688
21	fusA2	2	4
22	Rv2216	2	3
23	ctpI	1	5905

24	Rv2262c	1	5623
25	Rv3725	1	2566
26	Rv2885c	1	2506
27	lipF	1	1018
28	Rv0470A	1	961
29	vapB18	1	819
30	fadD11	1	727
31	mycP2	1	213
32	fadD29	1	19
33	Rv1179c	1	5
34	Rv3785	1	5
35	glpQ1	1	4
36	cyp128	1	2
37	Rv3094c	1	3
38	ctpC	1	1
**39**	**esxA**	**1**	**1**
40	ethA	1	1
41	fadE35	1	1
42	pknH	1	1
43	ptrBa	1	1
44	ptrBb	1	1
45	Rv0249c	1	1
46	Rv0393	1	1
47	Rv0397	1	1
48	Rv1264	1	1

Supplementary Table S8. Genes containing scar indels are listed and the number of distinct scar indel sets in each gene are identified.

Gene	Number of distinct scar indels in the gene [a]	Gene description [b]	Conserved domain(s) in the gene product [b]	Gene essentiality in *in vitro* cultures [c]	*In vivo* effect of gene-disruption
Rv0176	1	Mce associated transmembrane protein	Uncharacterized membrane protein YckC, RDD family	NE	Required for *in vivo* growth in C57BL/6J mice[5]
cyp128	1	cytochrome P450 Cyp128	Cytochrome P450	NE	-
espI	10	ESX-1 secretion-associated protein EspI	MinD-like ATPase involved in chromosome partitioning or flagellar assembly; P-loop containing Nucleoside Triphosphate Hydrolases	NE	Required for *in vivo* growth in C57BL/6J mice[5]
Rv2542	1	hypothetical protein	Alpha/beta hydrolase	NE	-
espB	1	ESX-1 secretion-associated protein EspB	−	GA	-
sigM	2	ECF RNA polymerase sigma factor SigM	RNA polymerase sigma factor SigM; Sigma-70 region 2	NE	-
eccE1	3	ESX-1 secretion system protein EccE1	type VII secretion protein EccE	GA	Required for *in vivo* growth in C57BL/6J mice[5]
pks12	2	polyketide synthase	enoyl reductase of polyketide synthase; beta-ketoacyl reductase (KR) domain of fatty acid synthase (FAS), subgroup 3, complex (x); Phosphopantetheine attachment site; Dehydratase; Acyl transferase; Enoylreductase; Ketoacyl-synthetase C-terminal extension; Rossmann-fold NAD(P)(+)-binding proteins	NE	Required for *in vivo* growth in C57BL/6J mice[5]
eccCa1	5	ESX-1 secretion system protein EccCa	type VII secretion protein EccCa; FtsK/SpoIIIE family	NE	Required for *in vivo* growth in C57BL/6J mice[5]
Rv0823c	1	tRNA-dihydrouridine synthase	putative TIM-barrel protein, nifR3 family; Dihydrouridine synthase-like (DUS-like) FMN-binding domain	GA	-

Rv0458	1	aldehyde dehydrogenase	Ralstonia eutrophus NAD+-dependent acetaldehyde dehydrogenase II-like	NE	-
Rv0045c	2	hydrolase	Pimeloyl-ACP methyl ester carboxylesterase; Alpha/beta hydrolase family	NE	-
fadE35	1	acyl-CoA dehydrogenase FadE35	Proteins involved in DNA damage response, similar to the AidB gene product; crotonobetainyl-CoA dehydrogenase	NE	-
Rv0397	1	hypothetical protein	−	NE	-
Rv0393	1	hypothetical protein	HNH nucleases; Domain of unknown function (DUF222)	NE	-
ptrBa	1	probable protease ii ptrbb [second part] (oligopeptidase b)	−	NE	-
aofH	2	flavin-containing monoamine oxidase	Monoamine oxidase; NAD(P)-binding Rossmann-like domain	NE	-
mycP2	1	membrane-anchored mycosin	type VII secretion-associated serine protease mycosin; Peptidase domain in the S8 and S53 families	NE	-
glpQ1	1	glycerophosphoryl diester phosphodiesterase	Glycerophosphoryl diester phosphodiesterase family	GA	-
Rv2561	5	pseudogene	−	NE	-
sppA	1	protease IV SppA	Signal peptide peptidase A (SppA) 67K type, a serine protease, has catalytic Ser-Lys dyad	NE	-
Rv0470 A	1	hypothetical protein	UbiA family of prenyltransferases (PTases)	GA	-
lipF	1	carboxylesterase LipF	Acetyl esterase/lipase; alpha/beta hydrolases	GA	-
ctpI	1	cation-transporter ATPase I	Magnesium-transporting ATPase (P-type); E1-E2 ATPase; Cation transporting ATPase, C-terminus; Soluble P-type ATPase	NE	-
eccD1	3	ESX-1 secretion system protein EccD1	type VII secretion integral membrane protein EccD	NE	Required for *in vivo* growth in C57BL/6J mice[5]

fusA2	1	elongation factor G	elongation factor G; domains similar to domain IV of the bacterial translational elongation factor (EF) EF-G; domains similar to the C-terminal domain of the bacterial translational elongation factor (EF) EF-G; Domain III of Elongation Factor G (EFG); Domain II of Elongation factor Tu (EF-Tu)-like proteins; P-loop containing Nucleoside Triphosphate Hydrolases	NE	-
ptrBb	1	probable protease ii ptrbb [second part] (oligopeptidase b)	−	NE	-
ctpC	1	manganese/zinc-exporting P-type ATPase	Cation transport ATPase; Soluble P-type ATPase; E1-E2 ATPase	NE	Required for *in vivo* growth in C57BL/6J mice[5]
esxA	1	ESAT-6 protein EsxA	Uncharacterized conserved protein YukE	NE	-
Rv3094c	1	hypothetical protein	Acyl-CoA dehydrogenase related to the alkylation response protein AidB	NE	-
Rv0249c	1	succinate dehydrogenase membrane anchor subunit	−	NE	Required for *in vivo* growth in C57BL/6J mice[5]
Rv1132	1	hypothetical protein	Transmembrane protein of unknown function (DUF3556)	NE	-
Rv2885c	1	transposase	Transposase; Putative transposase DNA-binding domain; Helix-turn-helix domain	NE	Required for *in vivo* growth in C57BL/6J mice[5]
Rv1264	1	adenylyl cyclase	Class III nucleotidyl cyclases; Adenylate cyclase, class 3; Adenylate cyclase regulatory domain	NE	-
pknH	1	serine/threonine-protein kinase PknH	Serine/Threonine protein kinases, catalytic domain; Catalytic domain of bacterial Serine/Threonine kinases, PknB and similar proteins; PknH-like extracellular domain	NE	-
cut1	1	cutinase	alpha/beta hydrolases	NE	-
Rv3725	1	oxidoreductase	NAD dependent epimerase/dehydratase family; Rossmann-fold NAD(P)(+)-binding proteins	GA	-
Rv1179c	1	hypothetical protein	Superfamily II DNA or RNA helicase; P-loop containing Nucleoside Triphosphate Hydrolases	NE	-

Rv2262c	1	hypothetical protein	−	NE	-
fadD29	1	long-chain-fatty-acid--AMP ligase FadD29	Fatty acyl-AMP ligase (FAAL); acyl-CoA synthetase	NE	-
Rv3785	1	hypothetical protein	−	GA	-
Rv2216	1	epimerase family protein	atypical (a) SDRs, subgroup 8; NAD dependent epimerase/dehydratase family enzyme	NE	-
ethA	1	monooxygenase EthA	Predicted flavoprotein CzcO associated with the cation diffusion facilitator CzcD; Rossmann-fold NAD(P)(+)-binding proteins	NE	-
fadD11	1	fatty-acid--CoA ligase FadD11	Long-chain fatty acid CoA synthetases and Bubblegum-like very long-chain fatty acid CoA synthetases; Long-chain acyl-CoA synthetase (AMP-forming)	NE	-
eccB1	2	ESX-1 secretion system protein EccB	type VII secretion protein EccB, Actinobacterial; Type VII secretion system ESX-1, transport TM domain B	NE	Required for *in vivo* growth in C57BL/6J mice[5]
Rv1575	1	phage protein	−	NE	-
ltp1	1	lipid-transfer protein	lipid-transfer protein; Thiolase domain associated with sterol carrier protein (SCP)-x isoform and related proteins; SCP-2 has multiple roles in intracellular lipid circulation and metabolism	NE	-
vapB18	1	antitoxin VapB18	Transcription regulator of the Arc/MetJ class	NE	-

a: Distinct scars that are found in all three studies considered here

b: Source: Gene annotations for *Mycobacterium tuberculosis* H37Rv from the Gene database of NCBI

c: Essentiality call for each gene from DeJesus et al, 2017. Key: Essential (ES), Essential Domain (ESD), Growth-Defect (GD), Non-Essential (NE), Growth-Advantage (GA), and Uncertain (for short empty genes).

Supplementary Table S9. Indels in the MIRU-VNTR loci of *M. tuberculosis* reference H37Rv. The coordinates of MIRU regions in H37Rv were extracted from Yasmin et al.[6] For two of the regions (denoted by *), the reverse primer did not exactly match the reference genome. Thus, these two regions were given an arbitrary length of 600 bp from the start coordinate (average length of the other 22 MIRU regions is 454 bp). A total of 73 unique indels were found in the MIRU-VNTR regions, only 10 of which were >5bp.

MIRU region	MIRU start coordinate	MIRU end coordinate	Number of unique indels in MIRU region
'MIRU-2'	153974	154436	2
'Mtub-04'	423924	424153	2
'ETR-C'	577193	577499	2
'MIRU4-(ETRD-1)*'	580540	581140	6
'MIRU-40'	802217	802579	3
'MIRU-10'	959893	960489	4
'MIRU-16'	1644052	1644677	3
'Mtub-21'	1955557	1955723	3
'MIRU-20'	2059312	2059855	6
'Qub-11b*'	2163714	2164314	4
'ETR-A'	2165244	2165601	2
'Mtub-29'	2347337	2347647	1
'Mtub-30'	2401747	2402016	1
'ETR-B'	2461303	2461551	6
'MIRU-23'	2531585	2532411	2
'MIRU-26'	2995830	2996400	7
'MIRU-27'	3006901	3007512	2
'Mtub-34'	3171398	3171635	3
'MIRU31-(ETR-E)'	3192002	3192610	1
'Mtub-39'	3690859	3691157	1
'Qub-26'	4052918	4053590	2
'Qub-4156'	4156592	4157238	5
'MIRU-39'	4348427	4349025	5

Supplementary Table S10. Size distributions of small indels > 5 bp. Note that small indels are defined as indels < 50 bp.

Indel size (bp)	No. of insertions (total=669)	No. of deletions (total=1966)
6	180	328
7	58	166
8	50	150
9	146	556
10	37	140
11	31	127
12	34	214
13	22	52
14	15	48
15	18	67
16	10	26
17	12	16
18	22	36
19	0	7
20	2	5
21	12	4
22	3	4
23	1	0
24	3	6
25	2	3
26	1	0
27	5	3
28	1	1
29	0	0
30	0	5
34	0	1
35	0	1
37	1	0
39	2	0
45	1	0

Supplementary Information References

1. Lipworth, S. *et al.* SNP-IT Tool for Identifying Subspecies and Associated Lineages of Mycobacterium tuberculosis Complex. *Emerging Infectious Diseases* **25**, 482-488 (2019).
2. Brites, D. & Gagneux, S. Co-evolution of Mycobacterium tuberculosis and Homo sapiens. *Immunol Rev* **264**, 6-24 (2015).
3. Zhang, H.T. *et al.* Genome sequencing of 161 Mycobacterium tuberculosis isolates from China identifies genes and intergenic regions associated with drug resistance. *Nature Genetics* **45**, 1255-U217 (2013).
4. Walker, T.M. *et al.* Whole-genome sequencing for prediction of Mycobacterium tuberculosis drug susceptibility and resistance: a retrospective cohort study. *Lancet Infectious Diseases* **15**, 1193-1202 (2015).
5. Sassetti, C.M., Boyd, D.H. & Rubin, E.J. Genes required for mycobacterial growth defined by high density mutagenesis. *Mol Microbiol* **48**, 77-84 (2003).
6. Yasmin, M. *et al.* Quick and cheap MIRU-VNTR typing of Mycobacterium tuberculosis species complex using duplex PCR. *Tuberculosis* **101**, 160-163 (2016).
