# Supplementary material for: Reversible gene silencing through frameshift indels and frameshift scars provide adaptive plasticity for Mycobacterium tuberculosis
Source: Nat Commun. 2021 Aug 4;12:4702. doi: 10.1038/s41467-021-25055-y (PMC8339072; doi:10.1038/s41467-021-25055-y)
Supplement: Supplementary file 3 — Description of Additional Supplementary Files [file 41467_2021_25055_MOESM3_ESM.pdf]

Description of Additional Supplementary Files

File Name: Supplementary Data 1
Description: LC scores at 20 sites upstream and downstream of orphan indel positions (Fig 2a and S3).

File Name: Supplementary Data 2
Description: LC scores at 20 sites upstream and downstream of non-indel positions (Fig 2a and S3).

File Name: Supplementary Data 3
Description: H scores at 20 sites upstream and downstream of orphan indel positions (Fig 2a and S3).

File Name: Supplementary Data 4
Description: H scores at 20 sites upstream and downstream of non-indel positions (Fig 2a and S3).

File Name: Supplementary Data 5
Description: Data for read-mapping quality at all indel positions (Figure S11).
