# Supplementary material for: Reversible gene silencing through frameshift indels and frameshift scars provide adaptive plasticity for Mycobacterium tuberculosis
Source: Nat Commun. 2021 Aug 4;12:4702. doi: 10.1038/s41467-021-25055-y (PMC8339072; doi:10.1038/s41467-021-25055-y)
Supplement: Supplementary file 9 — Reporting Summary [file 41467_2021_25055_MOESM9_ESM.pdf]

nature research

Corresponding author(s): David Alland

Reporting Summary

Nature Research wishes to improve the reproducibility of the work that we publish. This form provides structure for consistency and transparency in reporting. For further information on Nature Research policies, see Authors & Referees and the Editorial Policy Checklist.

Statistical parameters

When statistical analyses are reported, confirm that the following items are present in the relevant location (e.g. figure legend, table legend, main text, or Methods section).

n/a	Confirmed	
☐	☒	The exact sample size (*n*) for each experimental group/condition, given as a discrete number and unit of measurement
☐	☒	An indication of whether measurements were taken from distinct samples or whether the same sample was measured repeatedly
☐	☒	The statistical test(s) used AND whether they are one- or two-sided<br>*Only common tests should be described solely by name; describe more complex techniques in the Methods section.*
☒	☐	A description of all covariates tested
☒	☐	A description of any assumptions or corrections, such as tests of normality and adjustment for multiple comparisons
☐	☒	A full description of the statistics including central tendency (e.g. means) or other basic estimates (e.g. regression coefficient) AND variation (e.g. standard deviation) or associated estimates of uncertainty (e.g. confidence intervals)
☐	☒	For null hypothesis testing, the test statistic (e.g. *F*, *t*, *r*) with confidence intervals, effect sizes, degrees of freedom and *P* value noted<br>*Give P values as exact values whenever suitable.*
☐	☒	For Bayesian analysis, information on the choice of priors and Markov chain Monte Carlo settings
☒	☐	For hierarchical and complex designs, identification of the appropriate level for tests and full reporting of outcomes
☐	☒	Estimates of effect sizes (e.g. Cohen's *d*, Pearson's *r*), indicating how they were calculated
☐	☒	Clearly defined error bars<br>*State explicitly what error bars represent (e.g. SD, SE, CI)*

Our web collection on statistics for biologists may be useful.

Software and code

Policy information about availability of computer code

Data collection	Previously published data was used. The associated manuscripts are identified as references with PMID in the "Data Collection" section of online Methods.
Data analysis	All custom code was implemented in Python version 2.7 and was dependent on packages Numpy (version 1.14.1), SciPy (version 1.0.0), and matplotlib (version 2.1.2). The ScarTrek program is available at: https://github.com/aditi9783/ScarTrek. The FindingInfo program is available at: https://github.com/aditi9783/FindingInfo. Bioinformatics pipeline for analysis of WGS data is available at https://github.com/aditi9783/SNPTB. The remaining scripts for identifying evolutionarily independent isolates, for counting scar clades in phylogenetic trees, and for data analyses and data visualization are at https://github.com/aditi9783/Scar_manuscript_scripts. All code is publicly available under the MIT license.<br>All data analyses plots were generated using the matplotlib package (version 2.1.2). The phylogenetic trees were visualized using the Newick utilities package (version 1.6). The genome plot was drawn using Circos (version 0.69). Joyplots (Fig. S3) were generated using packages ggridges (version 0.5.0), ggplot2 (version 2.2.1), plyr (version 1.8.4), scales (version 0.5.0), and withr (version 2.1.2) on R (version 3.4.3).<br>The implementations of statistical tests in the SciPy package version 1.0.0 (scipy.stats) were used for computing the Pearson correlation coefficients (function 'pearsonr') and for performing the Welch t-tests (function 'ttest_ind') for comparing population means of distributions that have unequal variances.<br>The following software were used in this study: Trimmomatic (version 0.36), Bowtie2 (version 2.2.6), SAMtools (version 1.2) and BCFtools (version 1.2) for analysis of WGS data. GATK (version 4.0.8.1) HaplotypeCaller was used to detect indels for assessing ScarTrek performance. ART_Illumina, Q version 2.5.8 was used to generate simulated Illumina reads. Phylogenetic trees were then constructed

using MrBayes version 3.2.7a. Neighbor-Joining trees were generated using PHYLIP (Phylogeny Inference Package) version 3.696.

For manuscripts utilizing custom algorithms or software that are central to the research but not yet described in published literature, software must be made available to editors/reviewers upon request. We strongly encourage code deposition in a community repository (e.g. GitHub). See the Nature Research guidelines for submitting code & software for further information.

Data

Policy information about availability of data

All manuscripts must include a data availability statement. This statement should provide the following information, where applicable:

- Accession codes, unique identifiers, or web links for publicly available datasets
- A list of figures that have associated raw data
- A description of any restrictions on data availability

Data Availability
Data Collection. Whole genome sequencing data from 6509 clinical Mycobacterium tuberculosis isolates were collected from the following publications: Zhang et al., 2013, Nature Genetics (PMID:23995137); Walker et al., 2015, Lancet Infectious Diseases (PMID:26116186); Guerra-Assunção et al., 2015, Journal of Infectious Diseases (PMID:25336729). All clinical isolates were mapped to the M. tuberculosis H37Rv reference genome (Genbank ID: AL123456.3).
Source Data. Data underlying all figures has been provided in the accompanying “Source Data” file or the Supplementary Datasets 1-5.

Field-specific reporting

Please select the best fit for your research. If you are not sure, read the appropriate sections before making your selection.

☒ Life sciences ☐ Behavioural & social sciences ☐ Ecological, evolutionary & environmental sciences

For a reference copy of the document with all sections, see nature.com/authors/policies/ReportingSummary-flat.pdf

Life sciences study design

All studies must disclose on these points even when the disclosure is negative.

Sample size	We used previously published whole-genome sequencing data from 5,977 clinical isolates of Mycobacterium tuberculosis. We used all the published datasets that were available at the time of commencing this study and was the largest and most complete database of its type . We believed that the original sample size of 6,509 isolates (reduced to 5,977 isolates) was many times larger than needed to explore the contributions of in/dels and genome scars to on genomic plasticity in TB.
Data exclusions	WGS data from a total of 6,509 isolates were selected. After quality control analysis and mapping the data to the reference genome, only those isolates for which more than 50% of the raw reads mapped to the reference genome and average genome-wide coverage >20 were selected for further analysis. This resulted in a final WGS dataset of 5,977 isolates.
Replication	Replication was not performed because we studied one of the largest datasets available and our analysis showed us that all of the major phylogenetic clades of TB were well represented in the dataset used.
Randomization	Randomization was not performed because every sample in the data set was included in the analysis. The computational analysis itself is not amenable to randomization.
Blinding	The WGS data from clinical isolates that we analyzed was de-identified by studies that had published them and deposited them in databases. Thus, blinding was not necessary in this study. This type of data analysis is not subject to biases that might be introduced by the experimenter.

Reporting for specific materials, systems and methods

Materials & experimental systems

n/a	Involved in the study
☒	☐ Unique biological materials
☒	☐ Antibodies
☒	☐ Eukaryotic cell lines
☒	☐ Palaeontology
☒	☐ Animals and other organisms
☒	☐ Human research participants

Methods

n/a	Involved in the study
☒	☐ ChIP-seq
☒	☐ Flow cytometry
☒	☐ MRI-based neuroimaging
